# Quantitative Optical Nondestructive Evaluation of Bitumen–Aggregate Stripping Using Standardized Fluorescence Imaging and HSV Segmentation

**DOI:** 10.3390/s26175511

**Published:** 2026-08-30

**Authors:** Xuanliang He, Yi Peng, Yulin He, Lingyun Kong, Hongzhou Zhu, Huiying Mao, Junhao Zhai, Xianrui Liu, Yao Zhou

**Affiliations:** 1 School of Civil Engineering, Chongqing Jiaotong University, Chongqing 400074, China; hxl@mails.cqjtu.edu.cn (X.H.); konglingyun@cqjtu.edu.cn (L.K.); zhuhongzhouchina@126.com (H.Z.); 2 National and Local Joint Laboratory of Traffic Civil Engineering Materials, Chongqing Jiaotong University, Chongqing 400074, China; 3 Chongqing Key Laboratory of Intelligent Integrated and Multidimensional Transportation System, Chongqing Jiaotong University, Chongqing 400074, China; maohuiying@mails.cqjtu.edu.cn (H.M.); zjh@mails.cqjtu.edu.cn (J.Z.); liuxianrui@mails.cqjtu.edu.cn (X.L.); 4 Key Laboratory of New Technology for Construction of Cities in Mountain Area, Ministry of Education, Chongqing University, Chongqing 400045, China; 5 Department of Civil and Environmental Engineering, National University of Singapore, Singapore 117576, Singapore; yulin.he@nus.edu.sg; 6 School of Transportation, Chongqing Jiaotong University, Chongqing 400074, China; 7 College of Computer Science, Sichuan University, Chengdu 610065, China; yaozhou@scu.edu.cn

**Keywords:** optical nondestructive evaluation, fluorescence imaging, bitumen–aggregate interface, image segmentation, coating loss quantification, measurement sensitivity

## Abstract

Interfacial coating loss in bitumen-coated aggregates is difficult to quantify because the exposed aggregate regions are spatially heterogeneous, visually subtle, and sensitive to illumination and background conditions. This study develops a fluorescence-based optical nondestructive evaluation (NDE) framework for quantitative assessment of bitumen–aggregate stripping under standardized laboratory imaging conditions. A dataset of 5760 fluorescence images was collected from 120 physical BAP specimens representing 20 bitumen–aggregate combinations, with six viewing directions recorded for each specimen under eight acquisition environments. A lightweight no-reference image quality assessment model (LAR-IQA), together with visual inspection of shadow suppression and segmentation robustness, was used to select a reference acquisition protocol. The black-background, UV plus natural-light, glass-enclosure configuration provided stable contrast while reducing shadow interference. Three candidate segmentation methods were benchmarked against manually annotated reference masks, and the HSVSC algorithm achieved the best overall performance, with the highest mean Dice coefficient of 0.73. Using the standardized sensing–processing workflow, the image-derived stripping ratio distinguished material-dependent coating-loss behavior and revealed significant sensitivity to acquisition parameters. Aggregate type dominated the measured response under the tested conditions, with the mean stripping level ranked as limestone (3.94%) < quartz fine sandstone (4.17%) < basalt (4.95%) < granite (15.56%). ANOVA based on the 20 combination-level mean stripping ratios derived from 120 specimens, together with grey relational analysis, further indicated that, within the tested material set, aggregate-related descriptors were more strongly associated with the measured stripping variation than the selected bitumen descriptors. The proposed framework provides a repeatable, non-contact optical NDE route for converting subjective visual stripping assessment into image-derived quantitative measurement.

## 1. Introduction

Bitumen–aggregate systems are subjected to moisture, thermal, oxidative, and mechanical actions that can progressively weaken the interfacial bond between the binder film and the mineral aggregate surface [1,2,3,4,5,6]. When this bond is degraded, partial loss of the bitumen coating exposes aggregate regions and provides an observable manifestation of interfacial damage. From a nondestructive evaluation perspective, the central challenge is not only to rank material combinations, but also to obtain a repeatable and spatially resolved measurement of coating loss without relying on subjective visual judgement. Therefore, a quantitative optical NDE method that can convert visually complex stripping patterns into a reproducible image-derived index is needed for laboratory assessment of bitumen–aggregate interfacial durability.

A variety of methods have been developed to evaluate adhesion or anti-stripping performance at the bitumen–aggregate interface. Mechanical testing methods typically characterize interfacial bonding capacity directly or indirectly through pull-off, shear, or peeling tests [7]. Methods based on surface free energy and wetting theory can explain the interfacial interactions between bitumen and aggregate from a physicochemical perspective [8]. In addition, BBS (Binder Bond Strength) or pull-off-based methods have enhanced the quantitative characterization of interfacial adhesion under controlled conditions [9,10]. These approaches are valuable for evaluating bond strength or thermodynamic affinity, but they do not directly provide a pixel-level measurement of the exposed aggregate area after stripping [11,12,13]. As a result, they are not sufficient by themselves for optical NDE of spatially heterogeneous coating loss on aggregate particles.

The size of the bitumen film area on the BAP surface represents the ability of bitumen adhering to aggregate surface under the stripping action from vehicle wheels or moisture damage. The bitumen peeling test has the advantage of directly evaluating the adhesion between bitumen and aggregate phases with naked eye by visually observing the extent of bitumen film peeling [14]. Hence, the ASTM D3625 (American Society for Testing and Materials standards) [15] covers a rapid procedure for visually observing the loss of adhesion in uncompacted asphalt-coated aggregate mixtures due to the action of boiling water. Nevertheless, the reliability of such approaches is compromised by operator subjectivity, resulting in poor repeatability, limited objectivity, and insufficient quantitative precision.

Digital image analysis enables objective identification of bitumen coating loss regions, eliminating the subjectivity inherent in visual observation and facilitating the transition from qualitative visual judgment to quantitative measurement-based evaluation of stripping behavior [16,17]. Tayebali et al. [18], employed colorimetric devices to quantify coating quality of asphalt mixtures following boiling water tests. Cui et al. [19], combined digital image analysis with active adhesion assessment techniques to quantify bitumen coating retention on basalt and steel slag aggregates, while Arbabpour Bidgoli et al. [20], developed a registered image processing method to analyze moisture-induced coating detachment. However, conventional image processing methods face significant challenges when aggregate and bitumen exhibit similar coloration, hindering accurate boundary delineation. To overcome this limitation, Peng et al. [21], proposed a fluorescence-based tracing method that enables precise quantification of bitumen coating loss area through enhanced contrast between phases.

Subsequent research has begun to explore the utility and underlying principles of the fluorescence tracing method. For instance, studies have demonstrated its effectiveness when integrated with mechanical tests such as pull-off testing, providing a more comprehensive evaluation of interfacial adhesion [22]. The functional mechanism has also been investigated through multi-scale analysis, revealing that the method’s efficacy stems mainly from physical interactions, such as van der Waals forces and molecular diffusion, rather than chemical reactions [23]. Furthermore, initial explorations have shown that sensor parameters and environmental factors, such as temperature, moisture, and material aging, can significantly influence fluorescence tracing characteristics [24,25]. However, the transition from a promising contrast-enhancement principle to a robust optical NDE procedure remains incomplete. In particular, image acquisition conditions, segmentation accuracy, ground-truth validation, and measurement sensitivity must be addressed as part of a complete sensing–processing–measurement chain.

The summary of the representative studies and the present work in terms of fluorescence tracing (FT), stripping quantification (SQ), controlled optical conditions (CO), image quality assessment (IQA), ground truth (GT), adhesion assessment (AA), and fluorescence tracing mechanism (FTM) is shown in Table 1. It shows that earlier studies usually focused on only one or several parts of the workflow, whereas the present work integrates controlled imaging, environment selection, segmentation algorithm performance evaluation, and stripping quantification into a more complete technical framework.

In broader NDE and infrastructure inspection applications, optical sensing and image-based analysis have been widely used to identify surface defects, monitor material condition, and support automated decision-making. Recent pavement image studies show a transition toward intelligent, multi-source, high-precision analysis frameworks [27,28,29,30,31]. More broadly, reviews of thermal and optical NDE show that measurement reliability depends strongly on excitation mode, acquisition geometry, environmental control, and data-processing strategy [32,33,34], while computer-vision inspection in civil infrastructure has similarly evolved from visual feature extraction toward automated, quantitative, and data-driven condition assessment [35,36,37]. Nevertheless, stripping quantification is more demanding than ordinary distress localization because it requires pixel-level separation of residual bitumen, exposed aggregate, shadows, and specular reflections within small and irregular regions of interest.

To bridge these gaps, this study develops and validates a standardized fluorescence-based optical NDE framework for quantitative evaluation of bitumen–aggregate stripping. The work moves beyond proof-of-concept fluorescence tracing by integrating controlled image acquisition, no-reference image quality assessment, ground-truth-based segmentation validation, and statistical interpretation of the image-derived NDE index. The main objectives of this paper are as follows:

1. A standardized fluorescence-imaging protocol is established for optical NDE of coating loss under controlled acquisition conditions, and the reference environment is selected by jointly considering no-reference image quality, shadow suppression, and downstream segmentation robustness.
2. Three candidate segmentation strategies are comparatively evaluated using manually annotated fluorescence images, and an HSV-based color-constrained method (HSVSC) is identified as an interpretable solution for the present pixel-level NDE task.
3. The resulting image-derived stripping ratio is defined as a quantitative NDE index for coating-loss assessment and is used to analyze material-related differences among bitumen–aggregate combinations.
4. The sensitivity of the image-derived index to acquisition parameters and its association with selected aggregate and bitumen descriptors are examined to support physically informed interpretation of the measured stripping trends.

**Figure 1:**
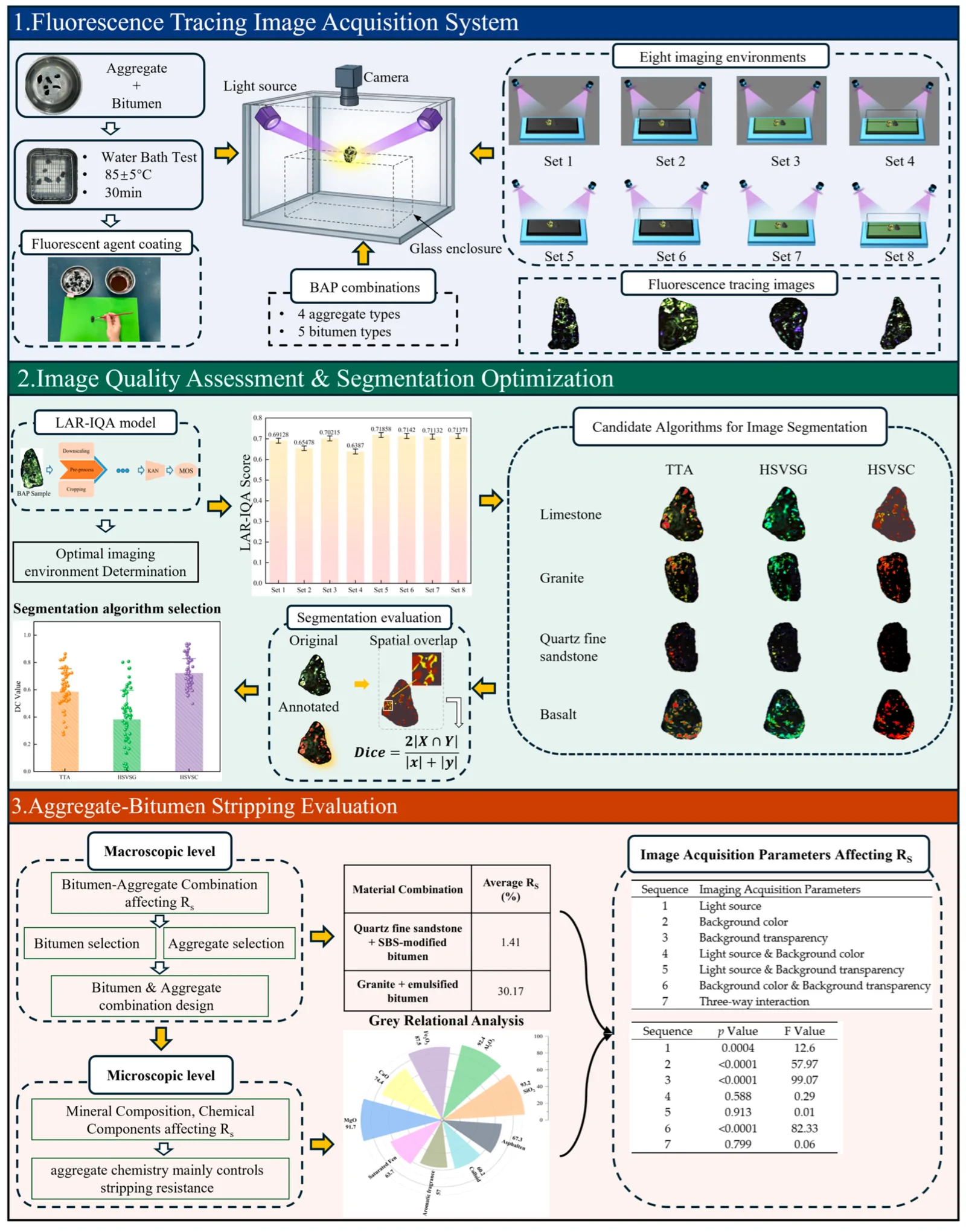
Workflow for this research.

To clarify the overall methodological route of the proposed optical NDE framework for quantitative bitumen–aggregate stripping assessment, the workflow is shown in Figure 1.

## 2. Materials and Sample Preparation

### 2.1. Properties of Aggregates and Bitumen

The aggregates selected for this research are granite, limestone, quartz fine sandstone, and basalt. They were oven-dried at 105 ± 5 °C for 4 h prior to testing. The basic chemical properties of the aggregates are listed in Table 2. Five bitumen types were used: 70# base bitumen (Sinopec, Beijing, China), 90# base bitumen (Sinopec, Beijing, China), SBR-modified bitumen (Sinopec, Beijing, China), SBS-modified bitumen (Sinopec, Beijing, China), and emulsified bitumen (Sinopec, Beijing, China). Their fundamental physical properties are summarized in Table 3, while their fractions (namely, Saturated Phen, Fragrant Phen, Gelatinous, Asphaltic) are listed in Table 4.

SBR-modified bitumen was prepared by modifying 70# base bitumen with rubber powder (derived from crushed waste tires) using high-speed shearing at 180 ± 5 °C. SBS-modified bitumen was produced by first blending the SBS modifier (3–6 wt%) with aromatic oil, then mixing it with 70# base bitumen under high-speed shearing (3000–5000 r/min, 170–180 °C, 1–2 h). Sulfur was added afterward to promote cross-linking stabilization.

The preparation of emulsified bitumen involved three stages: (1) dissolving a cationic emulsifier (e.g., quaternary ammonium salt, 0.3–3 wt%) in softened water (50–60 °C) and adjusting the pH to 2–5; (2) blending 70# base bitumen (120–140 °C) with the emulsifier solution (60:40 mass ratio) in a colloid mill, followed by high-speed shearing (3000–6000 r/min, 3–10 min) to achieve a uniform emulsion (1–10 μm particle size); and (3) cooling and storing the emulsion at room temperature.

### 2.2. Preparation of BAP Samples

The BAP samples were prepared following the bitumen stripping test outlined in ASTM D3625 (American Society for Testing and Materials standards) [15]. The specific procedure was as follows: dry aggregate was first weighed, and bitumen was added at 5.5 ± 0.2% by mass of the aggregate (e.g., 15.4 g of granite mixed with 1.07 g of bitumen). The mixture was then heated to 150–170 °C until the bitumen was fully melted. Under continuously maintained heating, the components were thoroughly blended to obtain a homogeneous bitumen and aggregate mixture, namely the BAP Samples.

Upon natural cooling to room temperature, the mixture was completely immersed in a temperature-controlled container maintained at 85 ± 5 °C for 30 min to complete the water bath test. During this period, any floating bitumen film was promptly removed to prevent re-adhesion. Then, the samples were taken out and cooled to room temperature again. Thus, the BAP samples with partially detached bitumen films are suitable for visual assessment of the bitumen stripping extent. The overall testing process is schematically illustrated in Table 5.

## 3. Fluorescence-Based Optical NDE Image Acquisition and Dataset Creation

### 3.1. Fluorescence-Based Optical NDE Image Acquisition System

**Figure 2:**
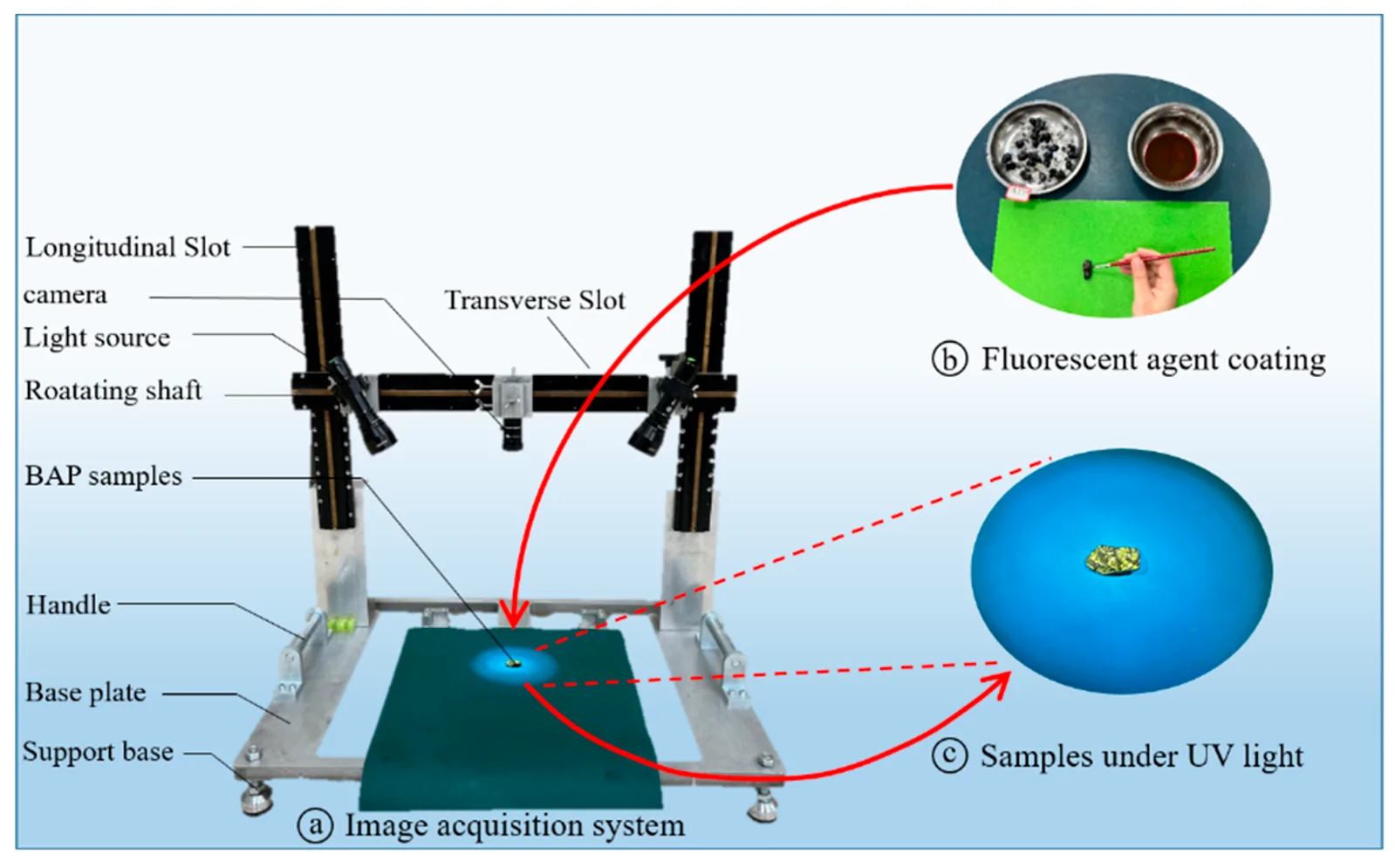
The fluorescence image acquisition illustration for BAP samples. (**a**) Image acquisition system; (**b**) Fluorescent agent coating; (**c**) Samples under UV light.

This method utilizes a self-developed fluorescence-based optical NDE image acquisition system, as shown in Figure 2a. The system consists of two ultraviolet light sources, one high-resolution camera, one tray, and one support base. The ultraviolet light sources are positioned on both sides of the camera to reduce projection-induced shadows. The light intensity is controlled at approximately 700 lux, and the camera lens is vertically focused on the object surface. First, the fluorescent agent is applied to the BAP samples, as shown in Figure 2b. The samples are then placed on the tray surface of the fluorescence tracing device. The two light sources project ultraviolet light onto the sample surface, and the fluorescence images of the samples are captured by the camera, as shown in Figure 2c. The imaging acquisition parameters are shown in Table 6.

Additional details of the fluorescent tracer, optical acquisition hardware, and key image-processing parameters are summarized in Appendix A, Table A1, to improve the reproducibility of the proposed standardized workflow.

### 3.2. Configuration of Image Acquisition Environments

Stratified random sampling was performed on all captured images to ensure data representativeness and improve the efficiency of subsequent image analysis. The experiment was conducted under eight imaging conditions, combining different light sources (Natural light and UV light), background colors (black and green), and background transparency (transparent with glass enclosure or opaque without glass enclosure). Then, the effective environment set to eliminate the shadow and reflection interference is determined by comparing the image quality. The abbreviations for each environment set are listed in Table 7, and the corresponding schematics are presented in Table 8.

### 3.3. Dataset Generation

The bitumen stripping ratio of BAP was found to vary with observation angle. To account for this, fluorescence tracing images were captured in six orthogonal directions (top, bottom, left, right, front, and rear) for each physical sample, and the stripping ratio was averaged across all perspectives. The experimental design comprised four aggregate types and five bitumen types, yielding 20 distinct BAP combinations. Six samples for each combination were prepared to verify the reproducibility of the tests. The traced images were captured in six directions for each physical sample, yielding 720 directional images per environment. Thus, the study generated 5760 fluorescence-tagged BAP images under eight distinct environmental conditions. The six directional stripping ratios of each specimen were averaged to obtain one specimen-level $R_{s}$. For the combination-level material comparison and the two-way ANOVA without replication, the six specimen-level $R_{s}$ values within each bitumen–aggregate combination were further averaged to obtain one combination-level mean.

**Figure 3:**
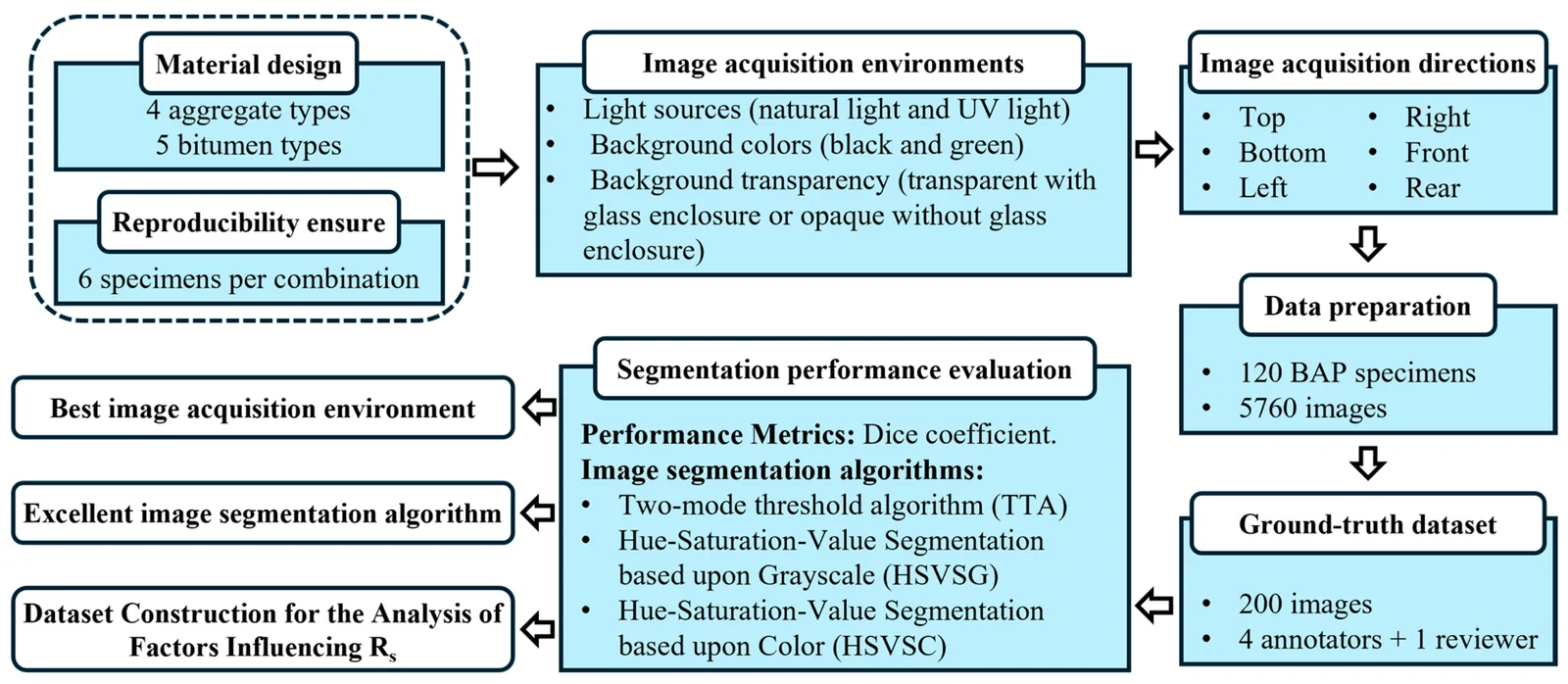
Sample-Image-Annotation-Analysis workflow.

To clarify the hierarchical relationship among raw dataset construction, the manually annotated subset, the optimal-environment image subset, and their respective downstream uses, the overall dataset construction and subset allocation workflow is illustrated in Figure 3.

## 4. Image Processing and Quantitative NDE Data Analysis Methodology

Upon adsorption of the fluorescent tracer, both the bitumen and aggregates of the BAP samples become lighter in color, thereby creating a clear contrast with the background. Thus, this study adopts the background extraction method proposed [21] to isolate BAP particles at first. Then, the fluorescence image sharpness evaluation and segmentation for bitumen stripping identification is performed as follows.

### 4.1. Image Quality Assessment

The LAR-IQA model, a lightweight no-reference image quality assessment model, was adopted to evaluate fluorescence image quality before image segmentation [38]. LAR-IQA was selected because its dual-branch architecture captures both global degradation and local artifacts while maintaining low computational cost [39]. In the present study, the LAR-IQA model was not used as a direct surrogate for stripping visibility or segmentation accuracy. Instead, it was used as one criterion for comparing acquisition environments. The final reference environment was selected by jointly considering no-reference image quality, shadow/interference suppression, and the downstream segmentation performance of the HSVSC method.

**Figure 4:**
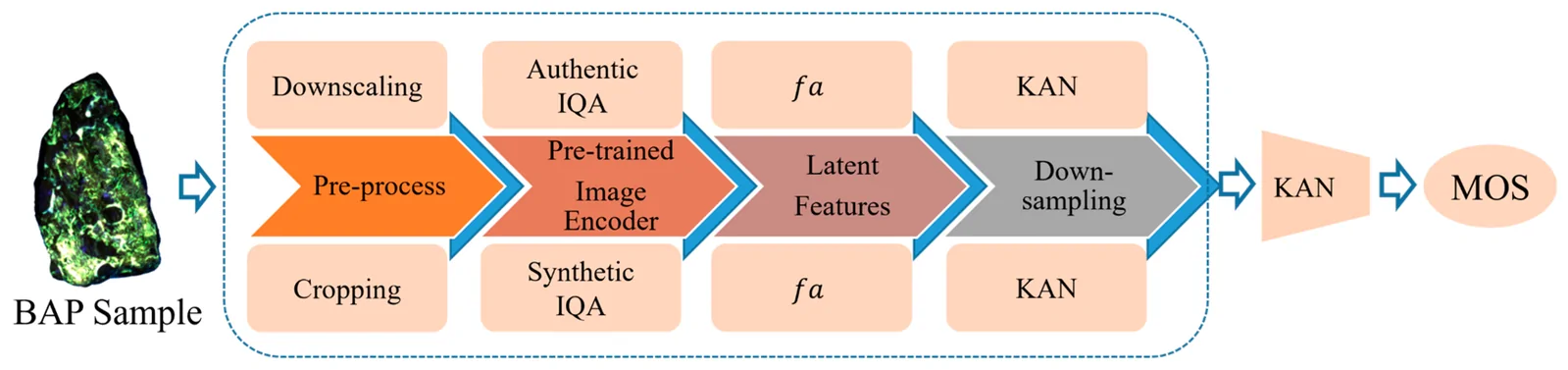
The operating scheme of the LAR-IQA.

The core execution strategy of the LAR-IQA model operates by two highly specialized, parallel branches after simultaneously feeding an input image into them, as shown in Figure 4: on one hand, it downscales the image to capture global authentic distortions like blur and noise, and on the other, it crops the image into patches to finely detect local synthetic artifacts such as compression effects. Subsequently, a shared Pre-trained Image Encoder extracts parallel Latent Features from these two streams. These features, representing global and local quality information respectively, are then fused after being refined by their individual KAN networks. Finally, this combined information is fed into a main KAN regression head to synthesize all the data and produce a final Mean Opinion Score (MOS) that quantifies the overall quality of the image. This image quality assessment framework ultimately assigns an image quality score to serve as an objective evaluation metric for the clarity of fluorescence images.

### 4.2. Fluorescence Tracing Mechanism and NDE Target Definition

**Figure 5:**
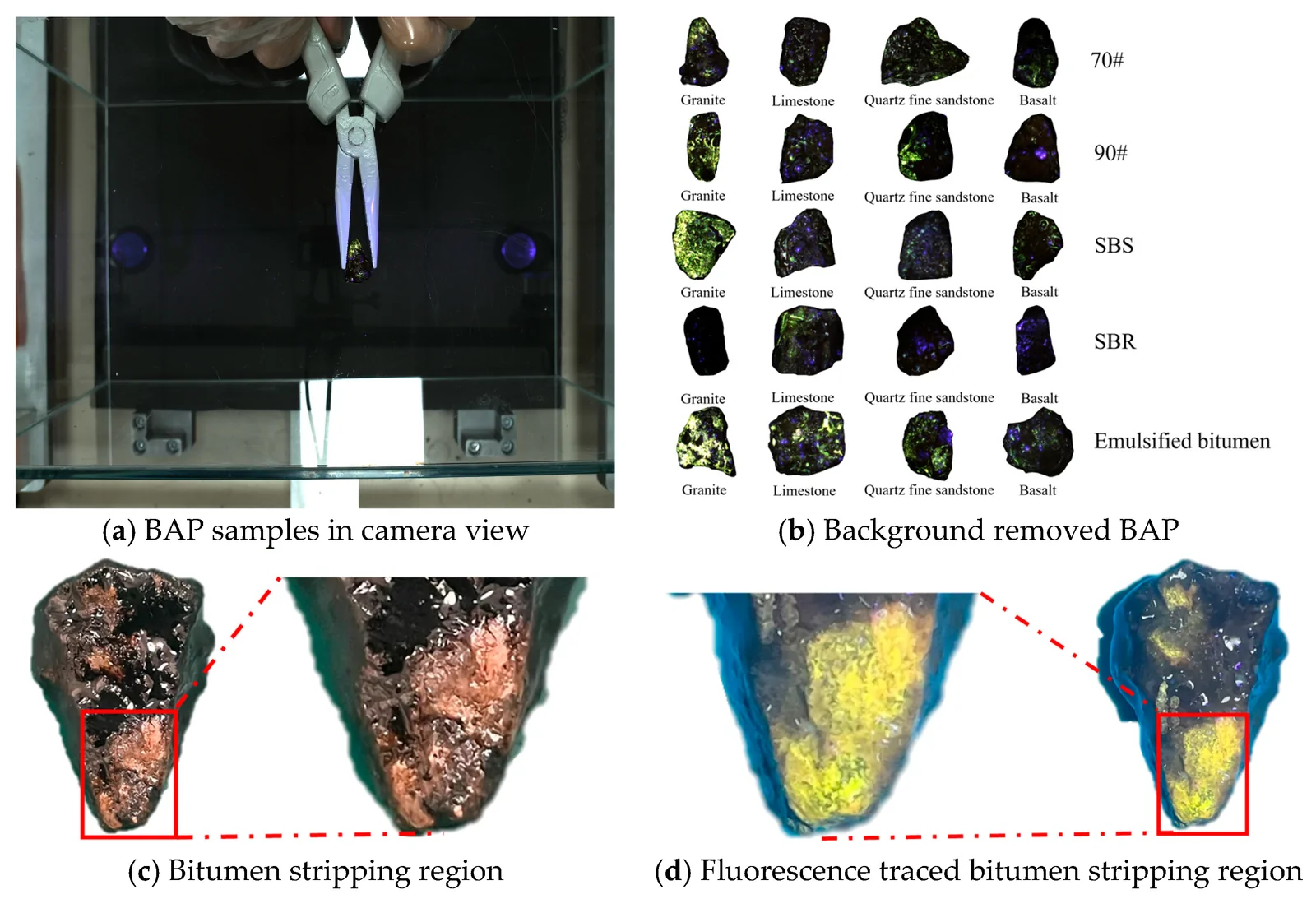
Representative fluorescence responses of bitumen-covered and aggregate-exposed regions under UV illumination.

Under UV illumination, the fluorescence tracer exhibits distinct responses on bitumen-covered and aggregate-exposed regions because of their different interactions with the tracer [12]. In the exposed aggregate region, the tracer remains on the surface and maintains a bright fluorescent response. In the bitumen-covered region, by contrast, the tracer is progressively absorbed into or mixed with the bitumen phase, and the visible fluorescence decays rapidly. As a result, the exposed aggregate tends to appear as a bright fluorescent region, whereas the bitumen-covered region appears relatively dark in the fluorescence image. Local blue-purple artifacts and specular reflections may also occur and must therefore be suppressed during segmentation. Figure 5a presents the BAP samples under the camera and the UV light, and Figure 5b shows the extraction of the BAP sample from the background. Figure 5c shows an enlarged view of the exposed region on the BAP surface in the original image, whereas Figure 5d shows the same region clearly highlighted by fluorescence.

### 4.3. Candidate Algorithms for Image Segmentation

The automated image segmentation is employed to identify the bitumen stripping area in this research (i.e., the exposed aggregate surfaces), as demonstrated in prior studies [21,25]. The fluorescence images were first evaluated and compared by three segmentation methods on: Two-mode threshold algorithm (TTA), a joint method combining Hue-Saturation-Value (HSV) and grayscale data, and HSV-based color segmentation. The best-performing algorithm from this comparison was then selected for the final analysis.

The general description of symbols and specifications for the image segmentation algorithms is as follows:

(1) The original color image is denoted as ***I_RGB_(x,y),*** where ***(x,y)*** represents the pixel coordinates, and the image size is ***m × n***;
(2) The grayscale image is denoted as I (x,y), and the grayscale level ranges from 0 to 255;
(3) The δ denotes the Kronecker delta function.

(1) $$\delta \left(a,b\right)=\left\{\begin{array}{cc} 1, & if\;a=b\\ 0, & otherwise \end{array}\right.$$

where a represents the current pixel, b represents the target pixel.

(4) The grayscale histogram is denoted as H(g), representing the number of pixels in the image with a grayscale value of g. which is defined as:

(2) $$H\left(g\right)=\sum_{X=1}^{M}\sum_{Y=1}^{N}\delta \left(I\left(x,y\right),g\right),g\in \left[0,255\right]$$

The subsequent thresholds K1 and K2 are both grayscale thresholds, with values ranging from 0 to 255. M represents the width of the image, and N represents the height of the image.

#### 4.3.1. Two-Mode Threshold Algorithm (TTA)

The **TTA** method utilizes the grayscale histogram of an image to identify the valley value between the primary peak and secondary peaks as the segmentation threshold, thereby separating brighter regions (potentially fluorescent area) from the background.

Given a grayscale range of g ∈ 0 to 255. The grayscale histogram H(g) is first computed. The grayscale value corresponding to the first global maximum in the histogram is identified (in the case of multiple maxima, the leftmost, i.e., minimum grayscale value is selected):

(3) $$g_{max1}=argmaxH\left(g\right),g\in [1,255]$$

**Figure 6:**
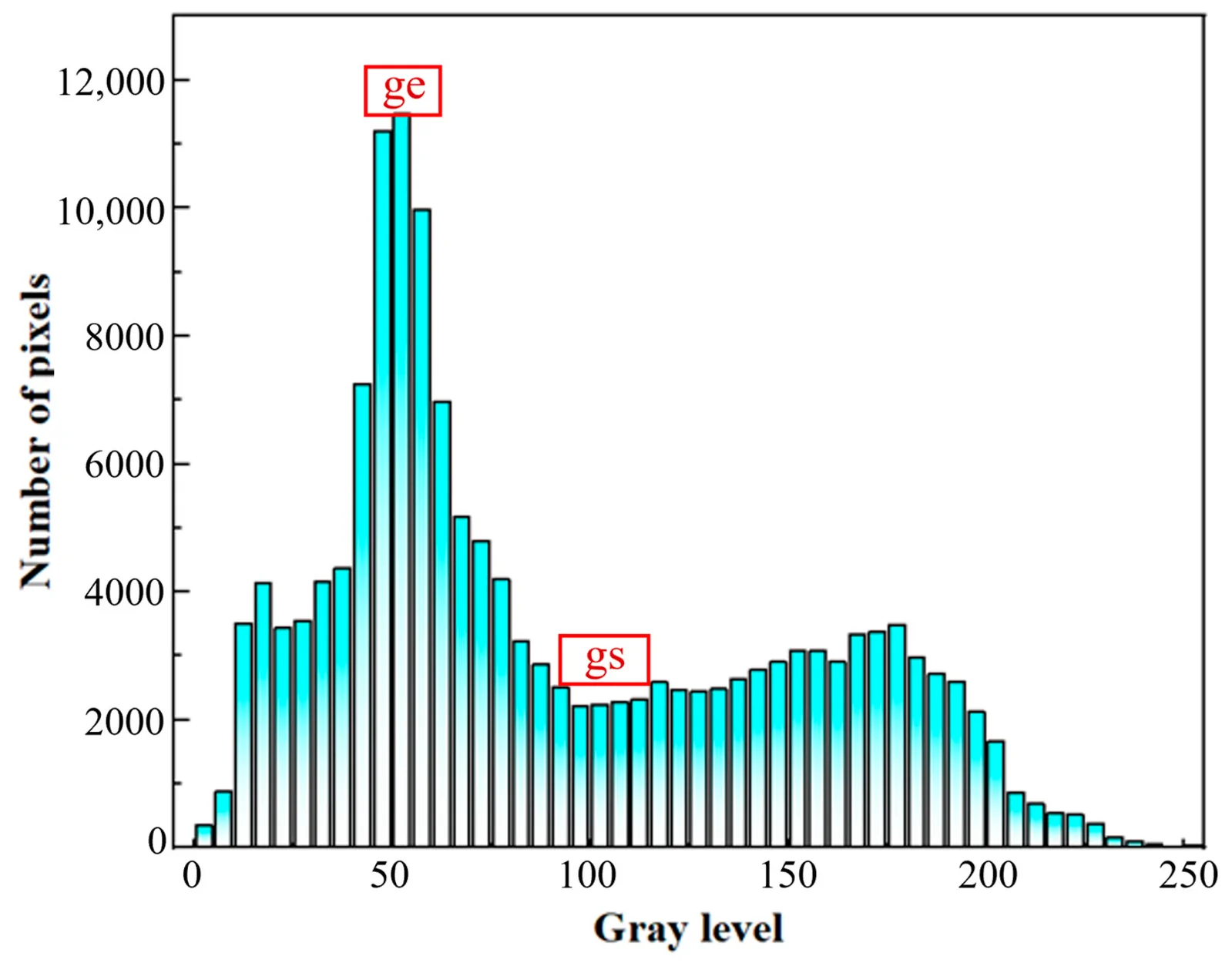
Gray-level histogram for threshold determination in the TTA method.

Within the interval $g>g_{max1}$ several local peaks (local maxima) are detected. These peaks are sorted in descending order according to their frequencies, and the grayscale values of the top 25 peaks [21] are selected, as shown in Figure 6. If fewer peaks are available, all detected local peaks are retained. Let $\left\{p1,p2,\ldots ,pk\right\}$ denotes the grayscale values corresponding to these local peaks, arranged in descending order of frequency. Determine the minimum grayscale value gs, and the maximum grayscale value ge.

(4) $$gs=min\left\{p1,p2,\ldots ,pk\right\},ge=max\left\{p1,p2,\ldots ,pk\right\}$$

For g**∈[**gs**,** ge**]**, the minimum value of ***H_(g),_*** denoted as ***K*_1_**, is selected as the optimal segmentation threshold for the fluorescence image:

(5) $$K_{1}=arg\;minH(g)g\in [gs,ge]$$

To define the upper threshold, ***K*_2_** is set to 255, such that all grayscale values greater than ***K*_1_** are treated as bright regions. The resulting binary mask ***M(x,y)*** is defined as:

(6) $$M(x,y)=\left\{\begin{array}{l} 1,if\;K_{1}<I(x,y)<K_{2}\\ 0,\;otherwise \end{array}\right.$$

Here, the concept of a mask M(x,y) is introduced. The mask M(x,y) is a binary image, where regions with M(x,y) = 1 correspond to fluorescent areas, while regions with M(x,y) = 0 represent non-fluorescent areas. In addition, $M_{color}(x,y)$ denotes a binary image defined based on color constraints.

#### 4.3.2. Hue-Saturation-Value Segmentation Based upon Grayscale (HSVSG)

The **HSVSG** method can be regarded as a **TTA**-based segmentation approach operating in the HSV color space. The core idea is to convert the **RGB** image into the **HSV** representation while preserving grayscale intensity information, and then combine **HSV**-based color constraints with grayscale-based thresholding to identify bitumen stripping regions.

In this context, **R**, **G**, and **B** denote the intensity values of the red, green, and blue channels, respectively. **H** (Hue) represents the color type, **S** (Saturation) describes the color purity, and **V** (Value) indicates the brightness level.

Specifically, **H** is used to distinguish fluorescent colors from background colors, **S** is used to separate colored pixels from grayscale pixels, and **V** is used to suppress non-fluorescent background information such as shadows and dark regions.

The implementation steps:

(1) Determine the color range of the fluorescence image. Identify the maximum value M and minimum value m of the RGB image in the fluorescence image:

(7) $$M=\max\left(R,G,B\right),m=min(R,G,B)$$

(2) Among the HSV components, saturation ***S*** is determined first. The saturation ***S*** of the fluorescence image is then calculated. When the differences among the **RGB** channel values increase, a particular color component becomes more prominent in the mixed light, thereby enhancing the pixel’s color purity. This effect is reflected as an increase in saturation, as shown in Formula (8).

(8) $$S=\left\{\begin{array}{c} \frac{M-m}{M},\;M\neq 0\\ 0,\;M=0 \end{array}\right.$$

(3) Determine the color type ***H*** of the fluorescence image. The hue ***H*** is calculated as shown in Equations (9)–(11).

If ***R*** is the maximum value:

(9) $$H=\frac{G-B}{M-m}*60{}^{\circ}$$

If ***G*** is the maximum value:

(10) $$H=\frac{B-R}{M-m}*60{}^{\circ}+120{}^{\circ}$$

If ***B*** is the maximum value:

(11) $$H=\frac{R-G}{M-m}*60{}^{\circ}+240{}^{\circ}$$

To determine which color a pixel belongs to, its position on the hue circle (0–360°) must be identified based on the maximum channel in RGB and the direction of deviation of the other two channels relative to the maximum channel. If the result exceeds 360° or is less than 0°, it is adjusted by subtracting from 360° to ensure it lies within [0°, 360°].

(4) Determine the brightness V of the fluorescence image. In the RGB color space, the overall brightness of a pixel is determined by its brightest channel. Therefore, the brightness ***V*** equals the maximum value ***M*** in **RGB**.

Brightness ***V*** is given by:

(12) V=M

(5) Construction of the color binary mask.

To associate color information with intensity characteristics, the HSV channels and the gray scale image were jointly analyzed in a pixel-wise manner, where each pixel shared the same spatial coordinates in both representations.

According to the literature [21], pixels with grayscale values g > 50 [25] were regarded as potential fluorescent regions.

The maximum and minimum hue values (H_max_ and H_min_) of these pixels were computed to determine the hue range of the fluorescent area. Based on this range, the color binary mask $M_{color}(x,y)$ was defined as shown in Formula (13).

(13) $$M_{color}(x,y)=\left\{h_{min}\leq h(x,y)\leq h_{max},s\left(x,y\right)\geq s_{min},v(x,y)\geq v_{min}\right\}$$

Among these, ***H_min_*** denotes the minimum hue value of the fluorescent region, ***S_min_*** represents the minimum saturation value, and ***V_min_*** indicates the minimum brightness value.

(6) Fluorescent Image Segmentation Based on HSV and Grayscale

The grayscale histogram ***H_(g)_*** was computed using the **TTA** method to determine $g_{max1}$. A grayscale threshold interval **[*K*_1_**, ***K*_2_**] was then defined in the vicinity of $g_{max1}$, yielding the grayscale mask ***M*_1_*(x,y)***. The final binary mask M(x,y) of the bitumen stripping area was obtained as the intersection of the color mask $M_{color}$ and grayscale mask M1(x,y):

(14) $$M(x,y)=M_{color}(x,y)\cap M_{1}(x,y)$$

#### 4.3.3. Hue-Saturation-Value Segmentation Based upon Color (HSVSC)

Due to the high adhesive properties of bitumen, impurities are easily attached to its surface, which may generate blue or purple noise under ultraviolet illumination. These noise regions can therefore be misclassified as bitumen-covered areas. The **HSVSC** approach first identifies pixels corresponding to bitumen black, converts blue and purple noise regions into bitumen-black pixels, and then directly extracts black regions to estimate the stripping rate.

The steps are as follows:

(1) Identify noise image regions. First, convert the image from the RGB color space to the HSV color space. Construct a binary function for the HSV values of noise pixels. B denotes the blue noise region, P denotes the purple noise region. As shown in Equations (15) and (16).

(15) $$B=\left\{\begin{array}{cc} 1,\; & if\;H\in [198{}^{\circ},\;245{}^{\circ}]\\ 0,\; & otherwise \end{array}\right.$$

(16) $$P=\left\{\begin{array}{cc} 1,\; & if\;H\in [245{}^{\circ},\;306{}^{\circ}]\\ 0,\; & otherwise \end{array}\right.$$

According to Kim et al. [40], the hue range for blue is [0.55, 0.68] (corresponding to [198°, 245°] on the color wheel), and for purple is [0.69, 0.85] (corresponding to [245°, 306°]).

The combined mask is defined as:

$$M_{combined}=B\cup P$$

(2) The pixels in the combined mask $M_{combined}$ were converted to black in the HSV space. In practice, black pixels were characterized by a zero saturation and a very low brightness, while the hue was set to zero for convenience. Then, extract pixels that meet the hue, saturation, and brightness of the black area. Generate a binary mask $M_{color}(x,y)$. The remaining area is the fluorescent traced region.

(17) $$M_{color}(x,y)=\left\{h_{min}\leq h(x,y)\leq h_{max},s\left(x,y\right)\geq s_{min},v(x,y)\geq v_{min}\right\}$$

### 4.4. Performance Metrics for NDE Algorithm Selection

(1) Segmentation performance evaluation metrics

The DC (Dice Coefficient) method is used to evaluate the performance of the image segmentation algorithms [41]. It was originally introduced by Lee Raymond Dice [42] and quantifies the consistency between the predicted results and the reference labels based on the proportion of spatial overlap between the segmented foreground region and the manually annotated foreground region.

It is defined by the following formula:

(18) $$Dice=\frac{2\left|X\cap Y\right|}{\left|X\right|+\left|Y\right|}$$

**Figure 7:**
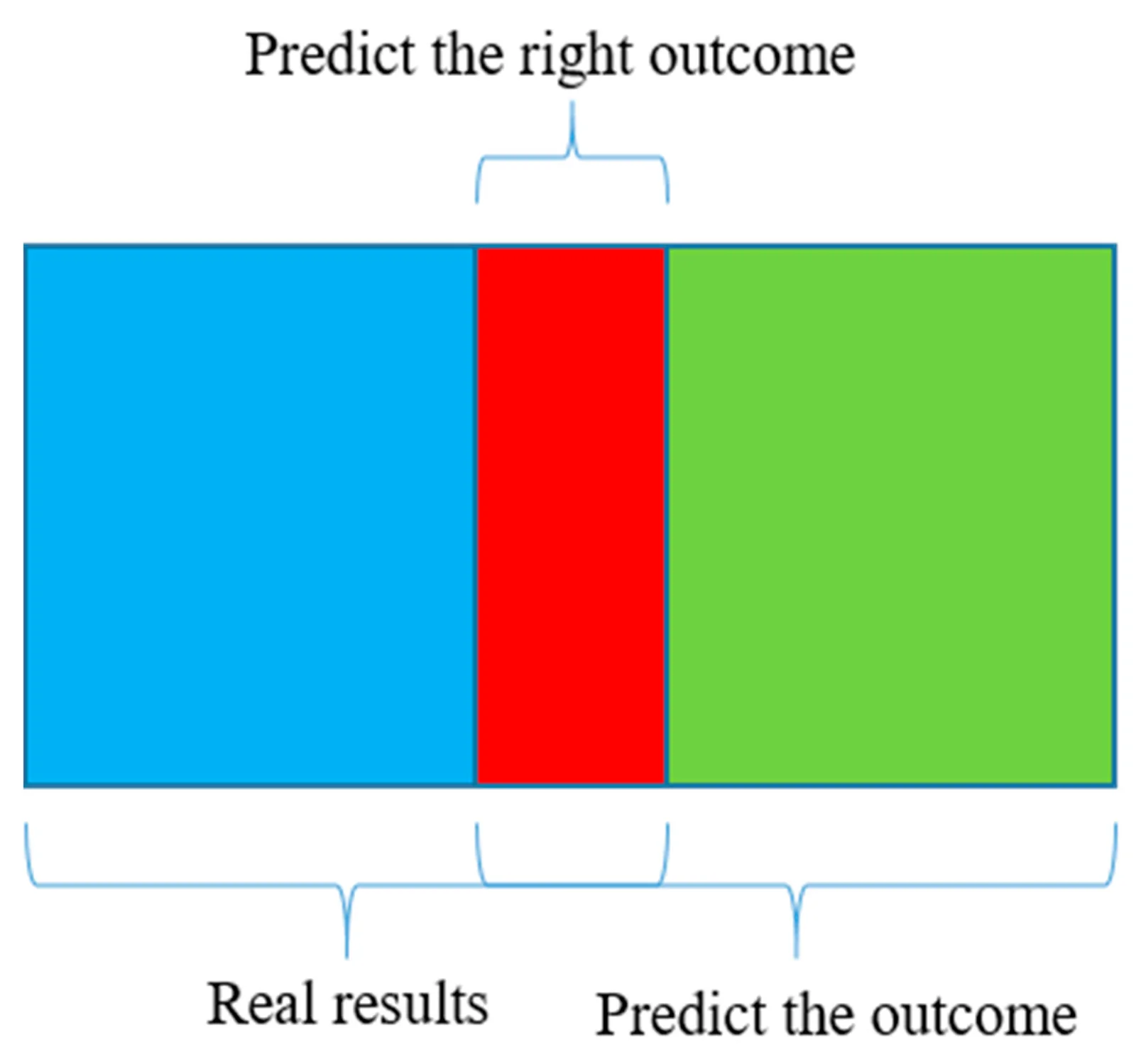
Illustration of the Dice coefficient principle.

Among them, **|X∩Y|** represents the number of pixels predicted as foreground and actually being foreground (True Positive, TP), while **|X|** and **|Y|** represent the total number of pixels in the predicted foreground area and the actual foreground area, respectively. A higher overlap between the real results and the prediction results in a higher **DC** value. The closer the coefficient is to 1, as shown in Figure 7, the more accurate the prediction is.

(19) $$IoU=\frac{A_{intersection}}{A_{union}}$$

(20) $$Precision=\frac{TP}{TP+FP}$$

(21) $$Recall=\frac{TP}{TP+FN}$$

where $A_{intersection}$ and $A_{union}$ denote the intersection and union areas of the predicted mask and the reference mask, respectively; TP, FP, and FN represent true positive, false positive, and false negative pixels, respectively.

(2) Bitumen stripping areas labelling framework

**Figure 8:**
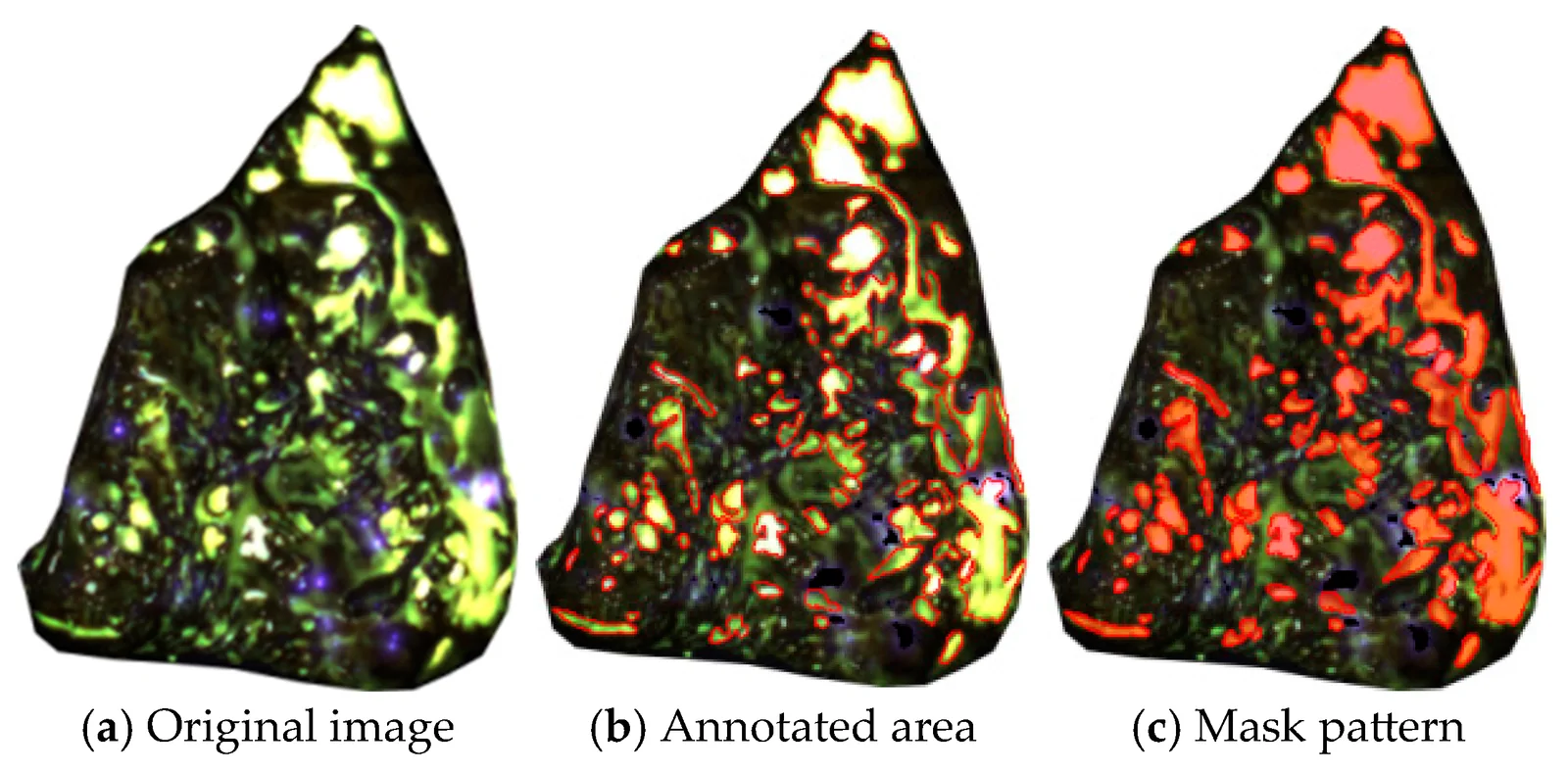
Image Labelling Workflow.

To establish a ground truth for evaluating image recognition algorithms, a subset of **BAP** images was manually annotated. This process involved three key steps:

(a) Background Separation: First, the **BAP** was isolated from the image background to define the region of interest, as shown in Figure 8a.
(b) Target Region Annotation: Next, using Adobe Photoshop^®^, the bitumen stripping areas within the BAP were manually outlined with pixel-level precision and marked in red, as shown in Figure 8b.
(c) Mask Generation and Refinement: Finally, a MATLAB^®^ (version R2024b; The MathWorks, Natick, MA, USA) script was employed to process the annotated images. The script extracted the red-labeled regions, converted them to the **HSV** color space for accurate color filtering, and then applied boundary refinement and region-filling techniques to generate a high-precision binary mask, as shown in Figure 8c.

Then, the final mask images were used as ground truth for evaluating the performance of the image segmentation algorithms.

For segmentation validation, a manually annotated subset of 200 images was constructed from the full dataset of 5760 fluorescence images. The selection adopted a stratified sampling strategy to ensure representation across the eight acquisition environments. Within each environment stratum, images were randomly drawn while checking the coverage of different bitumen–aggregate combinations and six viewing directions. The final subset was also screened to include images with varying stripping severities, so that both relatively mild and relatively severe exposed-area cases were represented in the annotation dataset.

The 200 image annotated dataset covered all eight acquisition environments, and a 60 image subset acquired under the selected reference environment (Set 6) was used for the final algorithm-performance evaluation reported in Section 5.2.

(3) Annotation consistency clarifying

To improve annotation reliability, the 200 selected images were distributed equally among four annotators, with each annotator labeling 50 images. All annotations were subsequently reviewed by a fifth annotator for quality control and final verification. When boundary ambiguity, weak fluorescence, or possible confusion with shadow/reflection artifacts was identified, the mask was re-examined and corrected according to the unified annotation criteria. This review-and-adjudication procedure was intended to reduce subjective bias and improve the consistency of the final ground-truth masks.

(4) Selection and evaluation of image segmentation algorithms

**Figure 9:**
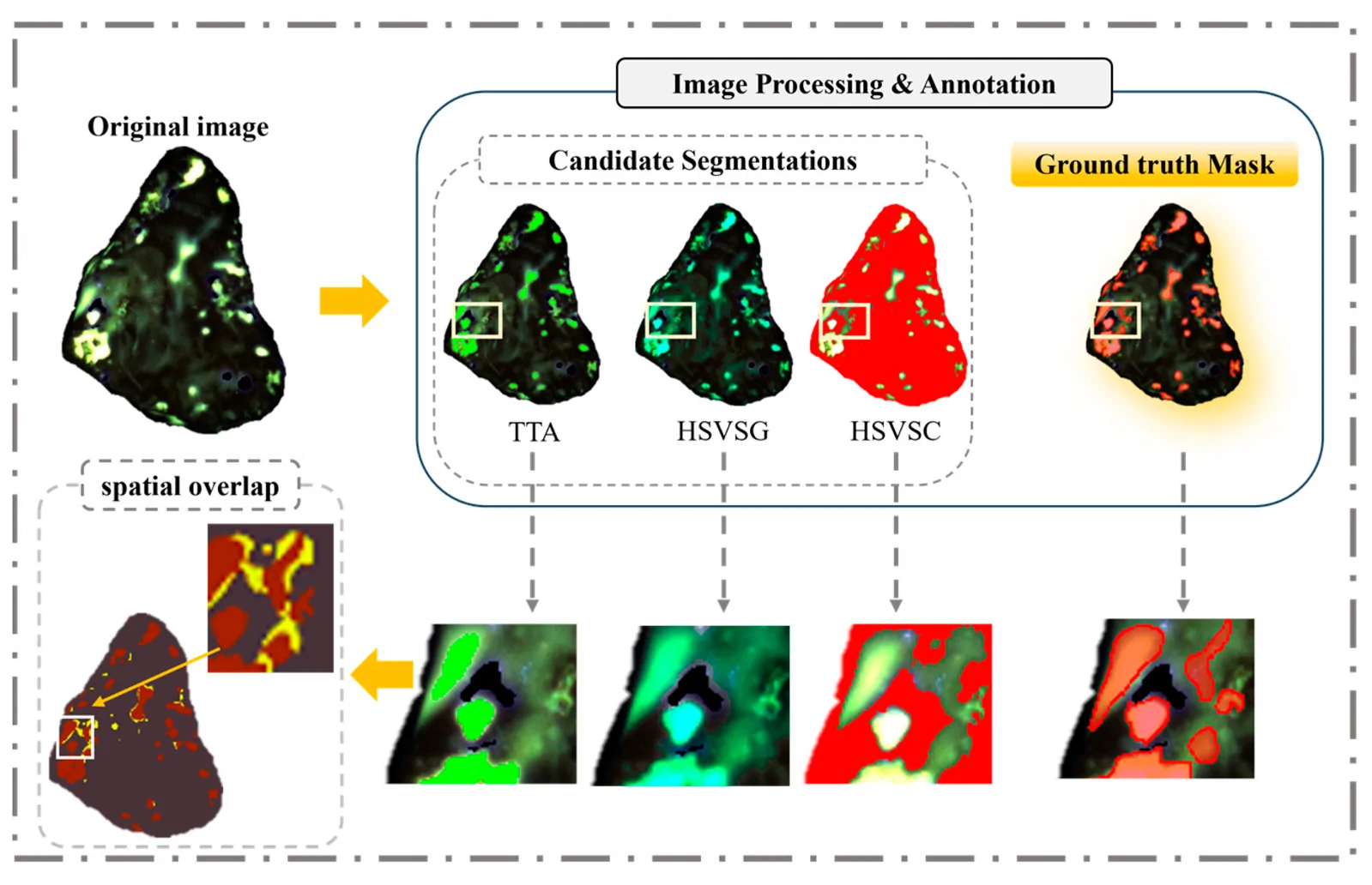
Framework for Segmentation Algorithm Evaluation and Selection.

To select the optimal model for segmenting bitumen stripping areas from several candidate algorithms, this study employed the DC value as the primary quantitative evaluation metric. This metric measures segmentation accuracy by calculating the degree of overlap between an algorithm’s segmentation result and the manually annotated “ground truth.” The evaluation and selection process was as follows:

(a) Fluorescence image segmentation: First, each candidate algorithm was applied to a sample set of images to detect stripped bitumen regions (i.e., exposed aggregate) and generate corresponding segmentation masks.
(b) **DC** calculation: Next, for each image in the sample set, the spatial overlap between the algorithm-generated mask and the manually annotated ground truth mask was computed using MATLAB^®^ to determine the **DC** value for that image. This process, visualized in Figure 7, involves calculating the overlap between the algorithm-generated mask and the manually annotated mask.
(c) Performance evaluation and decision: Finally, the mean **DC** value was calculated for each algorithm across the entire sample set. A higher mean coefficient signifies better consistency between the algorithm’s output and the manual annotations, indicating higher segmentation accuracy. The algorithm that achieved the highest mean **DC** value was selected as the optimal method for bitumen stripping region detection in this study, as shown in Figure 9.

### 4.5. Calculation of the Bitumen Stripping Ratio

The bitumen stripping ratio ($R_{s}$), defined as the percentage of aggregate surface area exposed after bitumen detachment, was determined using the selected image segmentation algorithm. The algorithm automatically delineated the bitumen-covered and exposed regions within fluorescence tracing images.

The stripping ratio for each sample was calculated using the following formula:

(22) $$R_{s}=\left(1-\frac{A_{\mathrm{covered}}}{A_{\mathrm{total}}}\right)*100\%$$

where $R_{s}$ is the bitumen stripping ratio; $A_{\mathrm{covered}}$ is the remaining bitumen coverage (i.e., the black pixels in the segmented image); $A_{\mathrm{total}}$ is the total surface area of the aggregate region.

## 5. Results and Discussion

### 5.1. Standardization of the Optical Acquisition Condition

**Figure 10:**
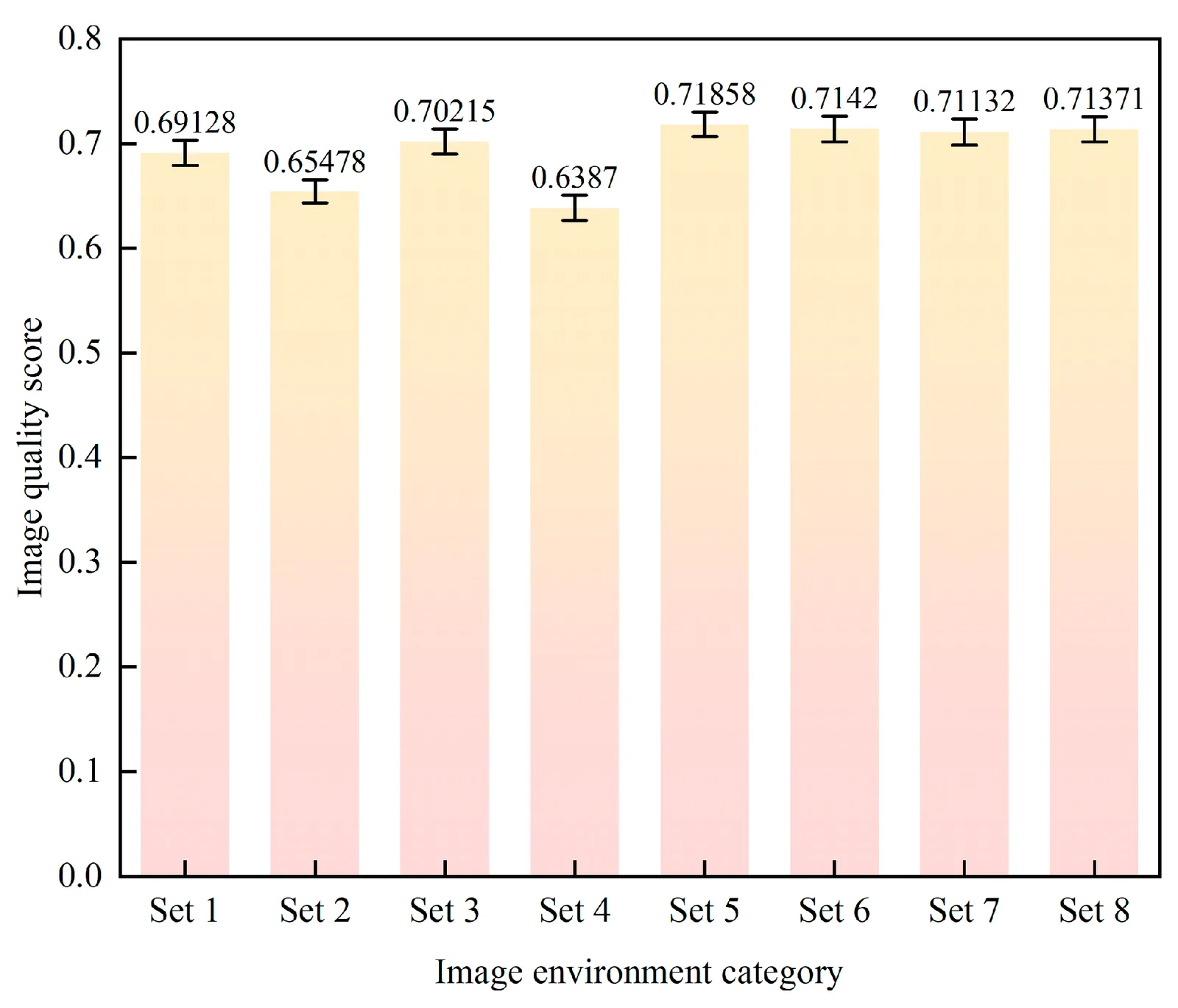
Comparison of Image Quality Scores under Different Acquisition Environments.

The quality of the acquired images is fundamental to the accuracy of the subsequent quantitative analysis of the bitumen stripping area. To determine the optimal acquisition conditions, this study conducted a systematic image quality assessment on 5760 images captured under eight distinct environmental configurations (detailed in Table 7 of the Methods section), with 720 images per environment. The LAR-IQA model was used for the assessment, and the results, showing the mean image quality scores and corresponding standard deviations for each environmental set (Set 1–Set 8), are presented in Figure 10.

To further reduce the subjectivity of environment selection, the downstream segmentation performance of the selected HSVSC method was additionally quantified for all eight acquisition environments. The corresponding Dice coefficients are summarized in Table 9.

A multi-variable comparative analysis of the data in Figure 10 and Table 9 leads to the following conclusions:

(a) Effect of Background Color: When other conditions were held constant, using a black background (B) consistently yielded a higher image quality score than a green background (G). For instance, Set 5 (B-UN, 0.7186) was significantly superior to Set 7 (G-UN, 0.7113), and Set 6 (B-UN-T, 0.7142) outperformed Set 8 (G-UN-T, 0.7137).
(b) Effect of Light Source: The use of a composite “UV + Natural light” source (UN) consistently produced better imaging results than a UV-only source (U). For example, the score of Set 5 (B-UN, 0.7186) was substantially higher than that of Set 1 (B-U, 0.6913), and Set 6 (B-UN-T, 0.7142) also scored higher than Set 2 (B-U-T, 0.6548).
(c) Effect of Background Transparency (Glass Enclosure): The image quality scores were slightly higher when no glass enclosure was used (background transparency = NULL). For example, Set 5 (B-UN, 0.7186) scored slightly higher than Set 6 (B-UN-T, 0.7142).

However, when the environments were further compared using the downstream segmentation performance of the HSVSC method, a different trend emerged. As shown in Table 9, Set 6 (B-UN-T) achieved the highest mean Dice coefficient (0.9085 ± 0.0125), followed by Set 5 (B-UN, 0.9031 ± 0.0062) and Set 3 (G-U, 0.9025 ± 0.0418). By contrast, Set 8 (G-UN-T) showed the lowest mean Dice coefficient (0.8087 ± 0.1996) together with the largest variability, indicating unstable segmentation performance under that condition.

Therefore, the reference acquisition environment was not selected based on the LAR-IQA score alone. Instead, it was determined by jointly considering no-reference image quality and downstream segmentation robustness. Although Set 5 (B-UN) achieved the highest LAR-IQA score, Set 6 (B-UN-T) provided the best overall performance because it combined a nearly equivalent image-quality score with the highest segmentation Dice coefficient and more stable suppression of shadow interference. Accordingly, Set 6 (B-UN-T), namely the black-background, UV + natural-light, glass-enclosure configuration, was selected as the reference image acquisition protocol for the remainder of this study.

### 5.2. Segmentation Validation Against Manual Reference Masks

**Figure 11:**
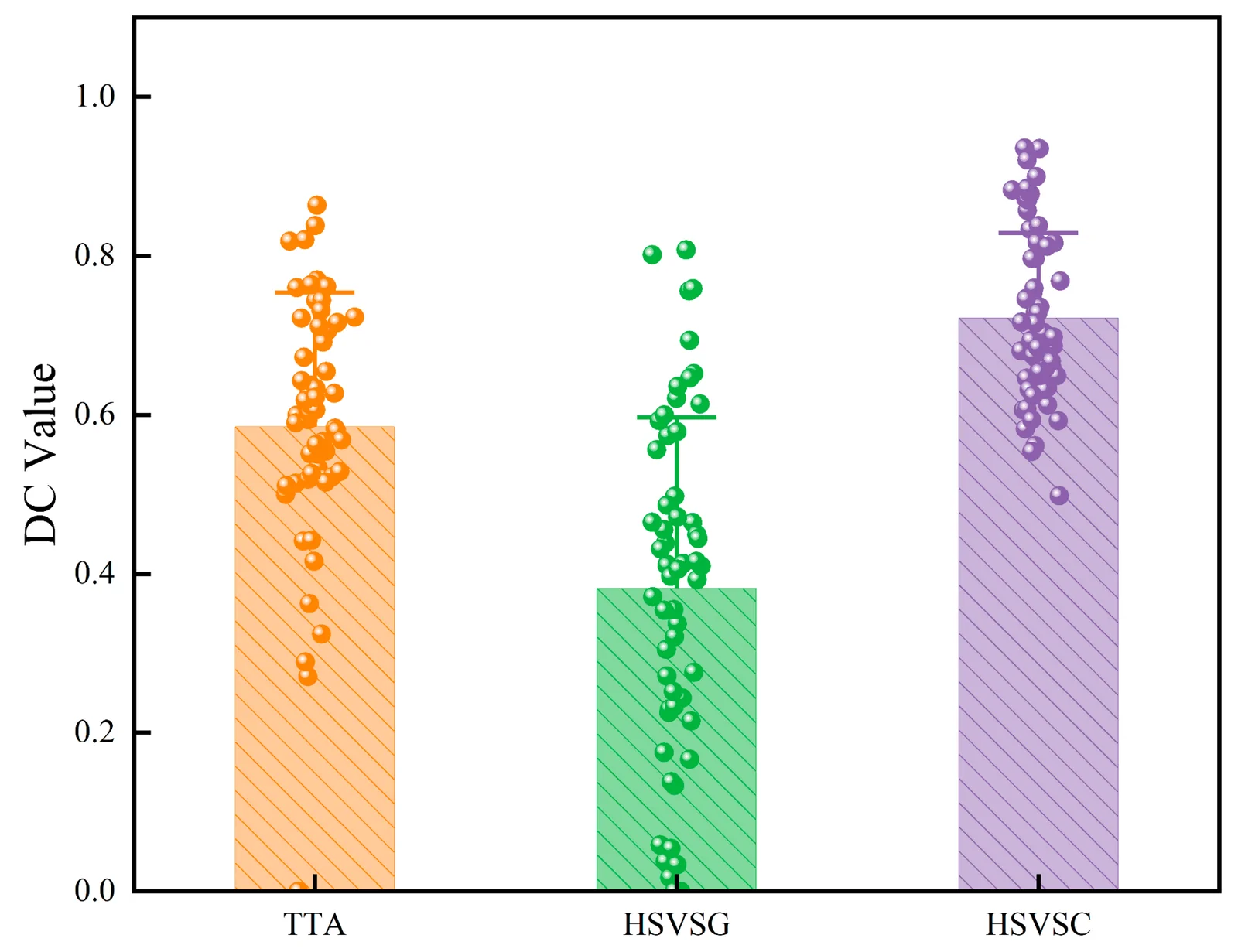
Performance Comparison of Different Image Segmentation Methods.

To accurately quantify the bitumen stripping area as an image-derived NDE index, this section presents a systematic performance evaluation of three candidate image segmentation algorithms, namely TTA, HSVSG, and HSVSC. To improve the robustness and representativeness of the evaluation, an annotated image dataset and a selected subset were used for different purposes. A manually annotated dataset of 200 images was constructed to cover all eight acquisition environments, material combinations, viewing directions, and stripping severities. From this dataset, a 60-image subset acquired under Set 6 (B-UN-T) was used for the final quantitative comparison of the candidate segmentation algorithms under the standardized imaging condition. The Dice coefficient (DC) was adopted as the primary metric to quantify the agreement between each algorithm-generated mask and the manual reference mask, while additional metrics, including IoU, Recall, and Precision, were further calculated together with their corresponding standard deviations and 95% confidence intervals to provide a more comprehensive assessment of segmentation performance. The detailed results are summarized in Table 10, and the Dice distributions are presented in Figure 11. Among the three methods, HSVSC achieved the best overall performance, with a mean Dice of 0.730, mean IoU of 0.575, mean Recall of 0.690, and mean Precision of 0.780, indicating superior segmentation accuracy and comparatively higher stability. By comparison, TTA showed moderate consistency with the manually annotated reference masks, whereas HSVSG exhibited the weakest performance and the greatest uncertainty. In Table 11, the three algorithms are compared on the same four representative fluorescence images. For TTA and HSVSC, the red regions denote the identified stripping areas, whereas for HSVSG, the green regions denote the identified stripping areas. The yellow regions represent the mismatch between the algorithm-generated mask and the manually annotated GT mask. Therefore, a smaller yellow region indicates better agreement with the GT. As shown in Table 11, HSVSC generally produces smaller mismatch regions than TTA and HSVSG, which is consistent with the quantitative results in Table 10.

The fundamental reason for the suboptimal performance of the TTA and HSVSG algorithms lies in their reliance on empirical thresholds, which are ill-suited for the complex and highly variable characteristics of the fluorescence images in this study.

The threshold search interval for the TTA is dependent on the 25 local peaks with the highest frequency—a fixed, empirical strategy. However, due to the diversity of aggregate and asphalt types and the varying intensity of the fluorescent response, the grayscale histogram patterns of the images fluctuate dramatically. This strategy is prone to incorporating noise, reflections, or background remnants into the calculation or ignoring true regional boundaries, thereby compromising the accuracy and stability of the determined threshold.

The HSVSG algorithm first applies a fixed grayscale threshold (g > 50) for pre-selection, which presumes a stable and sufficiently high brightness contrast between the target region and the background. In practice, however, some true stripped areas are incorrectly eliminated due to low grayscale values caused by a weak fluorescent response or surface texture occlusion. Concurrently, some non-target bright spots (such as reflections) are incorrectly retained. This “one-size-fits-all” empirical threshold cannot adapt to variations in materials and imaging conditions, severely undermining the method’s robustness.

In contrast, the HSVSC algorithm demonstrates superior performance by directly analyzing the target region’s color features (hue and saturation) in the HSV color space, rather than relying on unstable grayscale intensity. It suppresses blue-purple noise prior to region extraction, requires no training data, and operates with low computational cost. This approach effectively mitigates interference from uneven illumination, shadows, and material heterogeneity, enabling robust and precise identification of fluorescently marked regions. Moreover, its simplicity facilitates seamless integration into routine laboratory workflows, making it highly suitable for standardized engineering applications. The mean Dice coefficient of 0.73 indicates useful, but not error-free, agreement with the manually annotated reference masks. Potential failure cases include weak fluorescence, irregular stripping boundaries, small exposed regions, shadows, and specular reflections. False-positive segmentation may overestimate $R_{s}$, whereas false-negative segmentation may underestimate $R_{s}$. Therefore, small differences in stripping ratio should be interpreted cautiously, particularly for specimens with low stripping levels.

In short, given its accuracy, stability, and interpretability under the tested acquisition conditions, the HSVSC algorithm forms the core processing workflow used in the following NDE index calculation, material comparison, and statistical analysis. Deep-learning methods, such as U-Net, DeepLab, Mask R-CNN, and SAM, were not evaluated because they generally require larger annotated datasets, model training, and greater computational resources. In contrast, HSVSC requires no training, has low annotation and computational costs, and provides interpretable decision rules suitable for standardized laboratory use.

### 5.3. Application of the Image-Derived NDE Index to Material-Dependent Stripping Response

**Figure 12:**
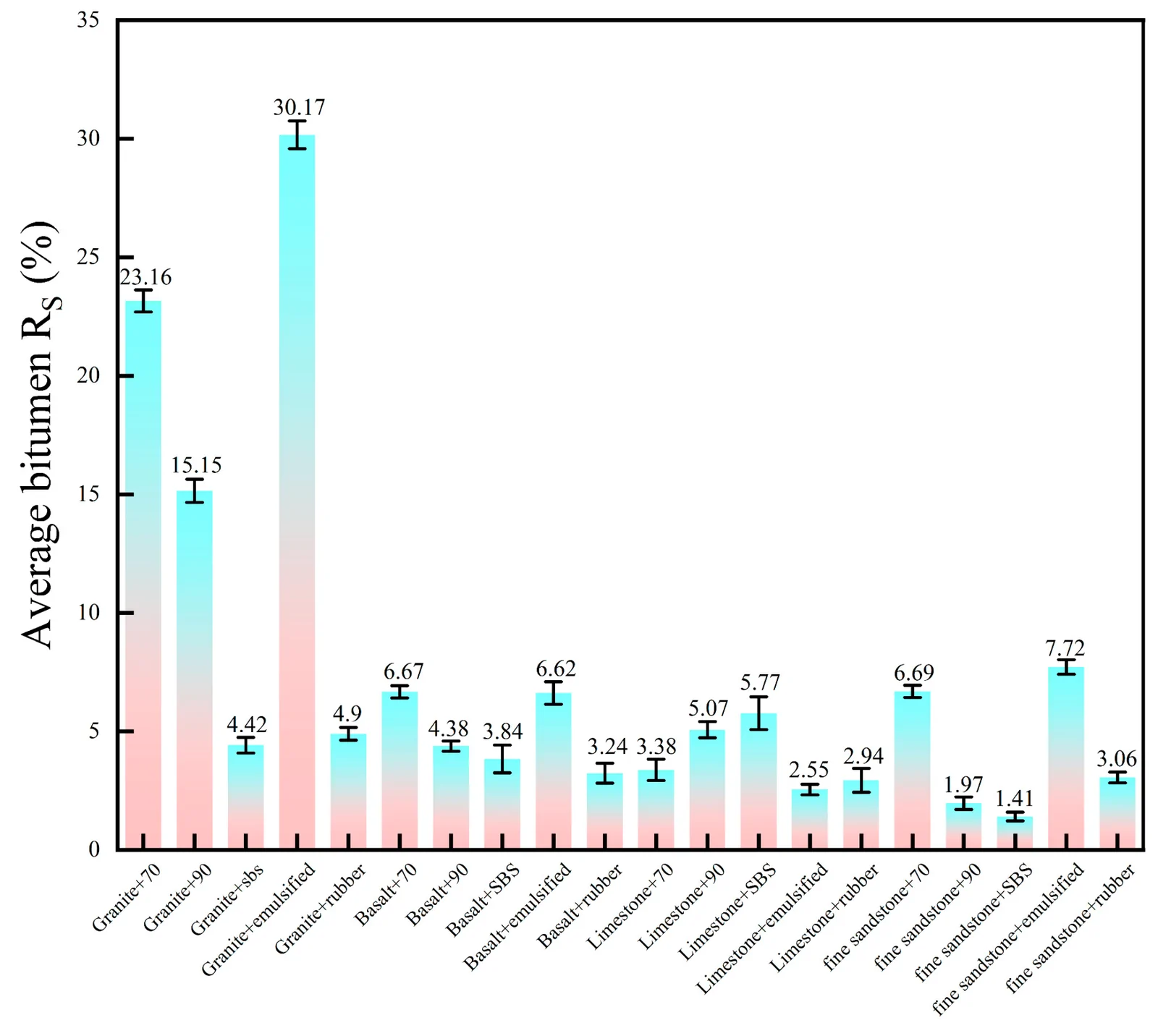
Average Stripping Ratio of Various Bitumen–Aggregate Combinations.

**Figure 13:**
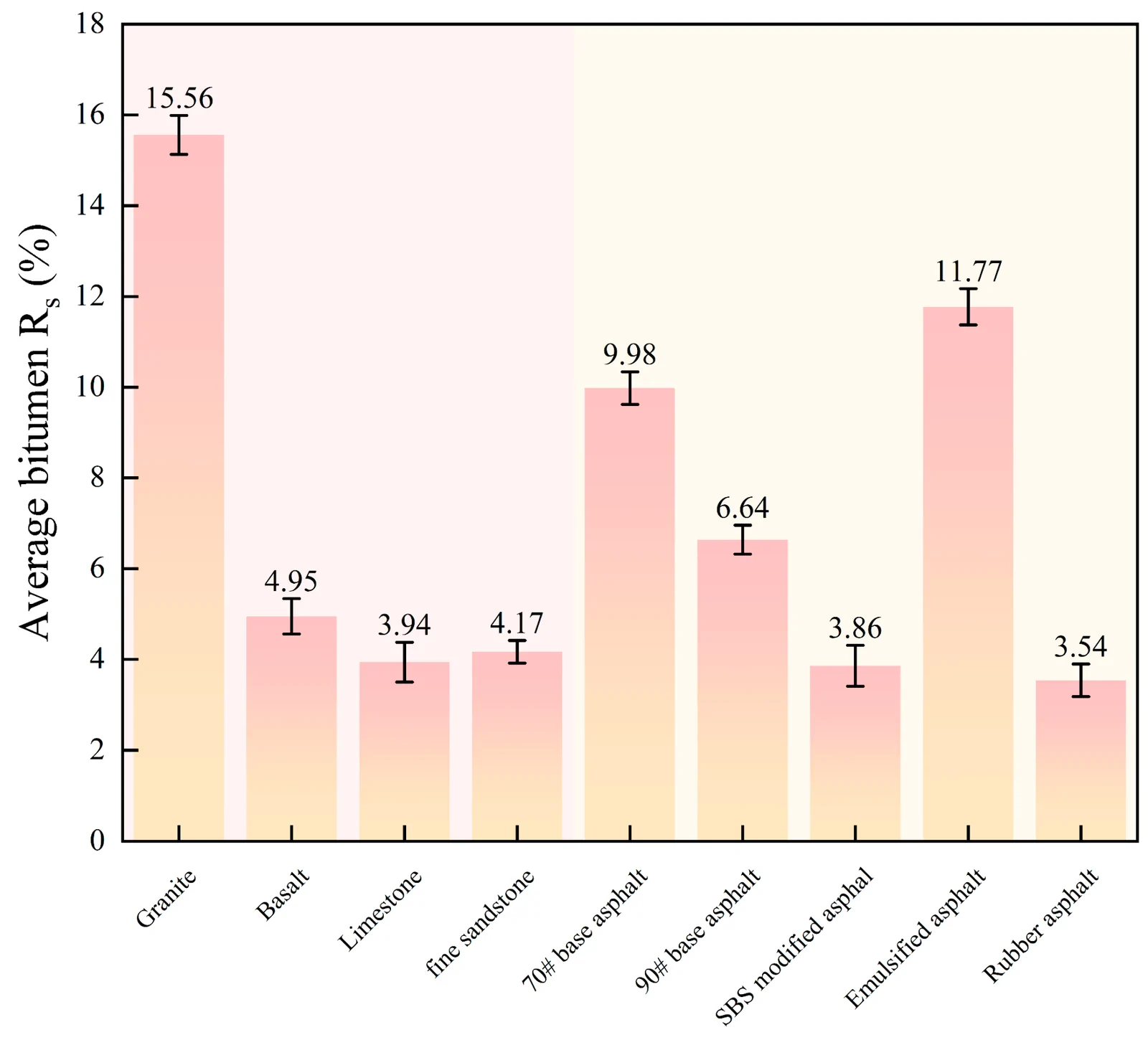
Average Stripping Ratio Classified by Aggregate and Bitumen Type.

Using the standardized image acquisition protocol established in Section 5.1 and the HSVSC segmentation method selected in Section 5.2, the $R_{s}$ was quantified for all 20 bitumen–aggregate combinations. Under this unified sensing and processing framework, the measured $R_{s}$ values showed clear material-dependent differences, as illustrated in Figure 12. The mean stripping ratios ($R_{s}$) and standard deviations for the 20 bitumen–aggregate combinations are shown in Figure 12. To clarify the individual effects of material type, aggregate and bitumen were analyzed as independent factors, and the corresponding mean stripping ratios are shown in Figure 13.

It is obvious that the combination of granite and emulsified bitumen yielded the highest stripping ratio by a significant margin ($R_{s}$ = 30.17%), representing the confluence of the most susceptible aggregate and the poorest-performing binder. In stark contrast, the most resilient pairings were fine sandstone with SBS-modified bitumen ($R_{s}$ = 1.41%) and fine sandstone with 90# base bitumen ($R_{s}$ = 1.97%), showcasing the benefits of a compatible aggregate-binder system.

A pronounced trend was observed among the four aggregate types in Figure 13. The average $R_{s}$, ranked from the highest to the lowest stripping resistance, was limestone (3.94%) < quartz fine sandstone (4.17%) < basalt (4.95%) < granite (15.56%). Limestone, a basic aggregate with a high CaO content (52.75%), exhibited the highest overall resistance to stripping, whereas granite, an acidic aggregate rich in SiO_2_ (69.61%), showed the most severe stripping. This trend is broadly consistent with the generally stronger adhesion between basic aggregates and the acidic polar groups in bitumen. Calcium-rich basic surfaces may promote favorable interfacial interactions, whereas silicate-rich acidic surfaces generally have a stronger affinity for water, allowing water molecules to compete for surface adsorption sites and facilitate displacement of the bitumen film. Consequently, basic aggregates tend to provide greater resistance to moisture-induced interfacial debonding, while acidic aggregates may be more susceptible to stripping [43]. Nevertheless, aggregate chemical composition alone may not fully explain the observed ranking, because other surface characteristics may also affect interfacial adhesion.

The choice of bitumen also influenced stripping resistance, although to a lesser extent than aggregate type. The average performance, ranked from best to worst anti-stripping capability, was: SBR-modified bitumen (3.54%) < SBS-modified bitumen (3.86%) < 90# base bitumen (6.64%) < 70# base bitumen (9.98%) < emulsified bitumen (11.77%). This result highlights the superior performance of polymer-modified bitumens (rubber and SBS), which enhance adhesion and toughness, compared to conventional base bitumens. Emulsified bitumen showed the weakest performance overall, likely due to the presence of emulsifying agents that can affect the interfacial bond.

From the viewpoint of method application, these material rankings demonstrate that the standardized fluorescence-imaging workflow can distinguish coating-loss levels among bitumen–aggregate combinations. The rankings should be interpreted as laboratory results obtained under the present conditioning and imaging protocol, rather than as universal design recommendations independent of mixture gradation, field aging, traffic loading, or environmental history.

### 5.4. Statistical Confirmation and Exploratory Chemical Interpretation

To verify the material-dependent trends described in Section 5.3, the six specimen-level stripping ratios for each material combination were first averaged. A two-way ANOVA without replication was subsequently performed using the resulting 20 combination-level mean stripping ratios, with aggregate type and bitumen type as the two factors (Table 12). The representative best- and worst-performing combinations are summarized in Table 13, where the range represents the difference between the maximum and minimum values among the material-combinations and is used to reflect the absolute variation in the indicator. The normalized range refers to the range after standardization and is used to compare the relative variation among different indicators. The coefficient of variation, defined as the ratio of the standard deviation to the mean, is used to evaluate the dispersion and stability of the indicator.

This statistical outcome is consistent with the ranking shown in Figure 12 and Figure 13. Granite produced substantially higher image-derived stripping ratios than the other aggregates, while limestone, quartz fine sandstone, and basalt remained in a relatively lower range. Although binder modification affected the mean level of the index, the aggregate substrate played the primary role in determining the overall coating-loss response in this short-term moisture-conditioning test. At the combination level, quartz fine sandstone plus SBS-modified bitumen showed the lowest mean together with relatively small variability, whereas granite plus emulsified bitumen produced the highest mean and the largest fluctuation. Although the main effect of bitumen type was not statistically significant, the combination-level results suggest that the improvement associated with modified bitumen may vary among aggregate types, reflecting differences in aggregate–bitumen compatibility and interfacial response. Because the aggregate × bitumen interaction was not formally evaluated, this material-dependent tendency should be regarded as a descriptive observation rather than a statistically confirmed mechanism. The chemical interpretation remains consistent with prior adhesion studies showing that surface polarity, adsorption capacity, and surface free energy are key determinants of adhesion durability [2,8,11,21,22].

**Figure 14:**
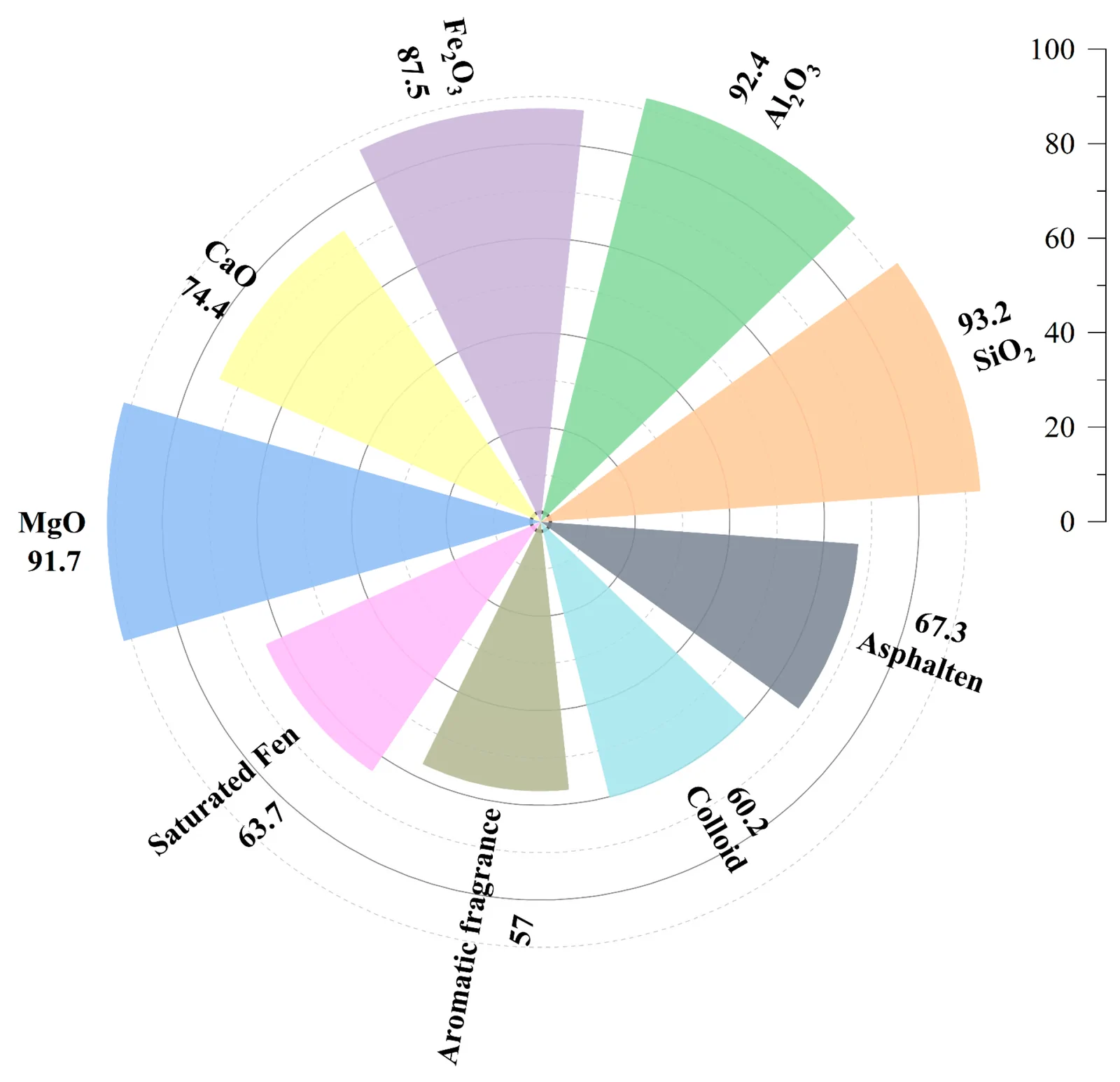
Ranking of Aggregate and Bitumen Chemical Components Influencing $R_{s}$.

To further identify the material factors associated with the measured coating-loss response [44], the GRA (grey relational analysis) was conducted using the measured stripping ratio as the reference sequence and the chemical components of aggregates and bitumen as comparison sequences. The results are presented in Figure 14.

.

It can be observed from Figure 14 that, among the aggregate components, ${SiO}_{2}$, ${Al}_{2}O_{3}$, and MgO showed the highest grey relational grades of 0.932, 0.924, and 0.917, respectively, followed by ${Fe}_{2}O_{3}$ (0.875) and CaO (0.744). These results indicate that aggregate-related descriptors were more strongly associated with stripping behavior than the selected bitumen descriptors in the present dataset. The relatively high grey relational grades of SiO_2_ and Al_2_O_3_ are broadly consistent with the observed sensitivity of silicate-rich aggregates to stripping under the present test conditions, whereas CaO and MgO may also be relevant to interfacial behavior.

For the bitumen fractions, the grey relational grades were ranked as follows: asphaltenes (0.673) > saturates (0.637) > resins/colloids (0.602) > aromatics (0.570). Although binder composition was associated with stripping performance, all four bitumen fractions showed lower relational grades than the major aggregate components.

Taken together, the ANOVA and GRA results provide a consistent interpretation: within the material set and conditioning protocol considered in this study, aggregate characteristics were more strongly associated with the variation in $R_{s}$ than the selected bitumen descriptors. However, this interpretation should remain cautious. Because the GRA was performed on a limited material set consisting of four aggregate types and five bitumen types, the resulting ranking reflects relative association within the present dataset rather than independent or causal material effects. In addition, the descriptors considered here do not fully capture other potentially important factors such as surface texture, porosity, morphology, or emulsifier-related effects. Therefore, the physicochemical interpretation should be regarded as exploratory rather than definitive.

In addition to these material-related effects, the measured $R_{s}$ may also be influenced by the acquisition parameters used to generate the fluorescence images. This measurement sensitivity is analyzed in Section 5.5.

### 5.5. Sensitivity of the Image-Derived NDE Index to Acquisition Parameters

While Section 5.1 established the reference acquisition condition used in the main analysis, it is also necessary to quantify how sensitive the image-derived NDE index is to changes in acquisition parameters. To address this issue, a three-way ANOVA was carried out using light source, background color, and background transparency as the experimental factors (Table 14).

The ANOVA results indicate that all three main factors significantly affected the measured $R_{s}$, namely light source (p=0.0004, F=12.6), background color (p<0.0001, F=57.97), and background transparency (p<0.0001, F=99.07). Among them, background transparency had the strongest effect, followed by background color. Only the interaction between background color and background transparency was significant (p<0.0001, F=82.33), whereas the interactions between light source and background color, between light source and background transparency, and the three-way interaction were not statistically significant. This finding suggests that the measured stripping ratio is particularly sensitive to the optical contrast created by the background configuration, because the enclosure and background condition jointly influence shadow suppression, edge continuity, and the separability of fluorescent and non-fluorescent regions. Specifically, background color and transparency can alter fluorescence-to-background contrast and introduce shadows or specular reflections, while changes in illumination can shift pixel brightness and color distributions, thereby causing false-positive or false-negative classifications under fixed segmentation thresholds. Therefore, standardized laboratory application requires consistent illumination, background configuration, camera geometry, and exposure settings, and stripping ratios obtained under different acquisition protocols should not be compared directly without calibration.

These results complement rather than repeat the analysis in Section 5.1. An operationally suitable acquisition protocol is identified in Section 5.1 for the main experiments, whereas the present section quantifies the extent to which deviations in acquisition conditions may alter the measured $R_{s}$. From a measurement perspective, this distinction is important. It indicates that $R_{s}$ should not be interpreted as a purely intrinsic material property when the imaging condition is not controlled. Instead, it is a sensing- and processing-dependent indicator whose reliability depends on the standardization of the acquisition environment.

Overall, Section 5.1, Section 5.2, Section 5.3, Section 5.4 and Section 5.5 demonstrate that the proposed framework can distinguish material-dependent stripping behavior only when the sensing and processing conditions are sufficiently controlled. In this sense, the quantitative reliability of the proposed optical NDE method relies on the combination of standardized fluorescence acquisition, robust image segmentation, and appropriate statistical interpretation.

### 5.6. Implications and Limitations for Optical Nondestructive Evaluation

The proposed method is a laboratory-scale optical NDE procedure for quantifying coating loss from bitumen-coated aggregate particles. Its principal contribution extends beyond fluorescence contrast enhancement to the establishment of a standardized measurement chain integrating fluorescence generation, controlled image acquisition, ground-truth-based segmentation validation, image-derived index calculation, and statistical interpretation. Unlike previous studies that generally addressed individual components of fluorescence-based stripping assessment, the present framework mitigates uncertainties associated with uncontrolled imaging conditions, unvalidated segmentation methods, and subjective interpretation. It therefore advances fluorescence-based stripping evaluation from a fragmented proof of concept toward a more repeatable and quantitatively interpretable optical NDE workflow.

Several limitations should nevertheless be acknowledged. First, the framework was developed under controlled laboratory conditions and cannot be directly transferred to field inspection. Variations in ambient illumination and imaging geometry may alter fluorescence contrast, while binder aging and surface contaminants, including dust, moisture, and oil, may affect tracer adsorption and introduce segmentation errors. Field implementation would therefore require controlled illumination, image calibration, surface-condition assessment, and validation using aged and contaminated pavement materials. Second, although the fluorescent tracer enhances phase contrast and enables quantitative coating-loss measurement, it does not identify the causal mechanism of interfacial failure. Combining the proposed method with independent adhesion or mechanical tests and multimodal characterization, particularly at progressive conditioning stages, could clarify the relationship between the image-derived stripping index and the underlying failure process. Third, although HSVSC outperformed the other tested algorithms, its mean Dice coefficient of 0.73 indicates moderate agreement with the reference masks and scope for further improvement through enhanced annotation protocols, three-dimensional surface information, or learning-based segmentation. Compared with recent learning-based segmentation studies in road engineering and optical NDE [27,28,29,30,31,32,33,34,35,36,37,45], direct numerical comparison should be made cautiously because of differences in imaging targets, datasets, annotation criteria, and acquisition conditions. The principal advantages of HSVSC remain its low computational cost, interpretability, and suitability for standardized laboratory implementation.

Measurement uncertainty may arise from illumination instability, variations in tracer application and fluorescence response, imaging geometry, segmentation errors, and manual reference-mask delineation. In this study, specimen-level standard deviations and confidence intervals describe measurement repeatability, the acquisition-condition ANOVA evaluates optical sensitivity, and the Dice/IoU metrics and multi-annotator review procedure assess segmentation performance and support annotation quality control. However, these uncertainty components were not propagated into a combined uncertainty interval. Accordingly, $R_{s}$ should be interpreted as a protocol-dependent estimate. Future work should establish a formal uncertainty budget through repeated imaging, illumination calibration, and quantitative inter-annotator agreement analysis.

## 6. Conclusions

This study developed and validated a standardized fluorescence-based optical NDE framework for the quantitative evaluation of bitumen–aggregate stripping. By integrating controlled image acquisition, no-reference image quality assessment, segmentation benchmarking against manual reference masks, and statistical interpretation, the study established an image-derived measurement route for laboratory coating-loss assessment. The main conclusions are as follows:

(1) Image acquisition conditions materially influenced both fluorescence image quality and downstream NDE quantification. Although the black-background, UV + natural-light configuration without a glass enclosure yielded the highest LAR-IQA score, the corresponding glass-enclosure setup was selected as the reference protocol because it effectively suppressed shadow interference and improved segmentation robustness.
(2) Among the three candidate segmentation methods, HSVSC provided the best overall performance for stripping extraction, achieving the highest mean Dice coefficient of 0.73 and showing more stable boundary recognition than TTA and HSVSG. This indicates that color-constrained segmentation is more suitable than fixed threshold-based approaches for the present fluorescence-based optical NDE images.
(3) Under the standardized sensing–processing protocol, the image-derived NDE index showed clear material dependence. Aggregate type had a stronger influence on the measured response than bitumen type, with the mean stripping level ranked as limestone (3.94%) < quartz fine sandstone (4.17%) < basalt (4.95%) < granite (15.56%). The most resistant and most vulnerable combinations were quartz fine sandstone incorporated with SBS-modified bitumen and granite incorporated with emulsified bitumen, respectively.
(4) Statistical analyses further indicated that, within the tested material set, aggregate-related descriptors were more strongly associated with stripping variation than the selected bitumen descriptors. Among the tested chemical descriptors, SiO_2_, Al_2_O_3_, and MgO showed relatively higher grey relational grades. However, these results should be interpreted as exploratory associations rather than evidence of causal or independent material effects.

Overall, the proposed framework moves bitumen–aggregate stripping assessment from subjective visual inspection toward repeatable and data-driven optical NDE. More importantly, the study contributes a workflow-level integration of controlled sensing, validated image processing, and statistical interpretation, thereby addressing the low repeatability, unstable segmentation performance, and fragmented methodological logic that have limited earlier fluorescence-based stripping studies. However, the present study remains limited to laboratory-scale samples and controlled optical conditions, and the chemical interpretation should be regarded as exploratory rather than causal. Future work should extend the framework to progressive damage states, more diverse material systems, and field-acquired images, and should further examine the linkage between image-derived stripping metrics and independent adhesion or NDE measurements.

## Figures and Tables

**Table 1:** Comparison of representative fluorescence-tracing-related studies and the present work.

Study	FT	SQ	CO	IQA	GT	AA	FTM
Cui et al. (2019) [19]	×	✓	△	×	△	×	×
Chen et al. (2020) [26]	✓	×	×	×	×	×	✓
Peng et al. (2023) [21]	✓	✓	×	×	△	×	△
Peng et al. (2024) [22]	✓	✓	△	×	△	✓	△
Zhao et al. (2025) [23]	✓	△	△	×	×	○	✓
Zhao et al. (2025) [24]	✓	△	△	×	×	○	✓

Note: “✓” denotes yes; “△” denotes limited; “○” denotes indirect evidence only; “×” denotes no.

**Table 2:** Basic Chemical Composition of the Selected Aggregates.

Attribute/Aggregate	Granite	Limestone	Quartz Fine Sandstone	Basalt
Main Ingredients	Quartz, feldspar	Calcite	Quartz	Pyroxene, olivine
SiO_2_ (%)	69.61	1.18	64.75	50.60
Al_2_O_3_ (%)	13.15	0.54	18.96	14.61
Fe_2_O_3_ (%)	3.5	0.32	3.8324	12.28
CaO (%)	3.18	52.75	3.0819	4.1
MgO (%)	0.0343	1.15	2.3652	0.15

**Table 3:** Basic Physical Properties of the Selected Bitumen.

Bitumen Name	Penetration (0.1 mm)	Ductility (cm)	Softening Point (°C)
70#	72.8	43	47.2
90#	84.3	>150	45.2
SBS	68.8	30.8	78.1
SBR	103.1	>150	46.5
Emulsified bitumen (4% residue)	62.1	1167	51.51

**Table 4:** The four fractions of the Selected Bitumen.

Bitumen Name	Saturated Phen (%)	Fragrant Phen (%)	Gelatinous (%)	Asphaltic (%)
70#	18.659	41.590	34.502	5.248
90#	20.671	35.129	39.073	5.009
SBS	23.462	36.134	30.731	9.605
SBR	20.545	30.555	40.910	7.928
Emulsified bitumen (4% residue)	18.659	41.590	34.502	5.248

**Table 5:** The bitumen partially detached BAP samples preparation scheme.

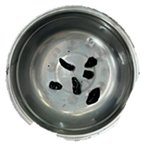	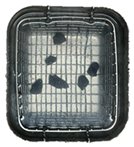	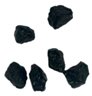
(**a**) BAP preparation	(**b**) Water bath test	(**c**) Bitumen partially detached BAP

**Table 6:** The imaging acquisition parameters.

Parameter	Exposure Time (Unit: ms)	Focal Length (Unit: mm)	Working Distance (Unit: cm)	Aperture Level (f-Stop)	Horizontal UV-Camera Distance (Unit: cm)
Value	100,000	8	44	6	30

**Table 7:** Abbreviations for the environmental set.

No.	Abbreviation	Background Color	Light Source	Background Transparency
Set 1	B-U	Black	UV	NULL
Set 2	B-U-T	Black	UV	YES
Set 3	G-U	Green	UV	NULL
Set 4	G-U-T	Green	UV	YES
Set 5	B-UN	Black	UV + Natural light	NULL
Set 6	B-UN-T	Black	UV + Natural light	YES
Set 7	G-UN	Green	UV + Natural light	NULL
Set 8	G-UN-T	Green	UV + Natural light	YES

Note: “YES” indicates the use of a glass enclosure as the sample stage, resulting in a transparent background. “NULL” indicates no glass enclosure, leading to an opaque background.

**Table 8:** Environment set schematics.

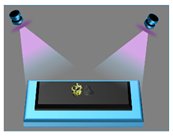	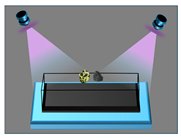	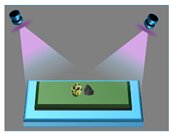	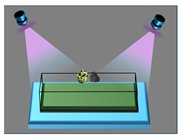
(**a**) B-U	(**b**) B-U-T	(**c**) G-U	(**d**) G-U-T
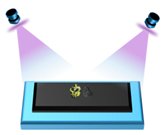	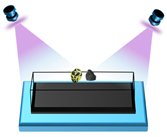	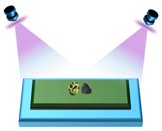	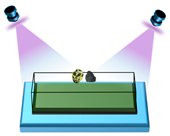
(**e**) B-UN	(**f**) B-UN-T	(**g**) G-UN	(**h**) G-UN-T

**Table 9:** Quantitative comparison of the eight acquisition environments based on HSVSC segmentation performance.

No.	Abbreviation	HSVSC Dice (Mean ± Std)
Set 1	B-U	0.8789 ± 0.0147
Set 2	B-U-T	0.8991 ± 0.0264
Set 3	G-U	0.9025 ± 0.0418
Set 4	G-U-T	0.8964 ± 0.0015
Set 5	B-UN	0.9031 ± 0.0062
Set 6	B-UN-T	0.9085 ± 0.0125
Set 7	G-UN	0.8935 ± 0.0412
Set 8	G-UN-T	0.8087 ± 0.1996

Note: To evaluate differences among acquisition environments, the Dice coefficients were calculated from matched images of the same specimens and viewing directions acquired under the eight environmental conditions, with all other acquisition parameters held constant.

**Table 10:** Quantitative performance comparison of the three segmentation algorithms.

Algorithm	Dice (Mean ± Std, 95% CI)	IoU (Mean ± Std, 95% CI)	Recall (Mean ± Std, 95% CI)	Precision (Mean ± Std, 95% CI)
TTA	0.554 ± 0.190, [0.506, 0.602]	0.383 ± 0.170, [0.340, 0.426]	0.620 ± 0.200, [0.569, 0.671]	0.500 ± 0.210, [0.447, 0.553]
HSVSG	0.315 ± 0.200, [0.264, 0.366]	0.187 ± 0.150, [0.149, 0.225]	0.380 ± 0.240, [0.319, 0.441]	0.270 ± 0.180, [0.224, 0.316]
HSVSC	0.730 ± 0.170, [0.687, 0.773]	0.575 ± 0.160, [0.535, 0.615]	0.690 ± 0.170, [0.647, 0.733]	0.780 ± 0.190, [0.732, 0.828]

**Table 11:** Visualization Comparison of Segmentation Results from Different Methods on Various Aggregate Types.

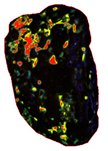	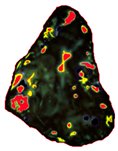	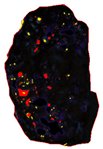	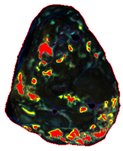
TTA VS. Granite	TTA VS. Limestone	TTA VS. Quartz fine sandstone	TTA VS. Basalt
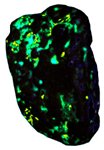	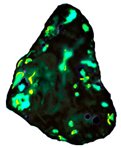	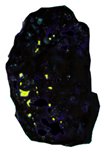	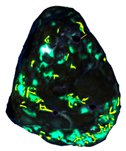
HSVSG VS. Granite	HSVSG VS. Limestone	HSVSG VS. Quartz fine sandstone	HSVSG VS. Basalt
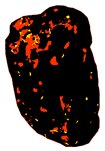	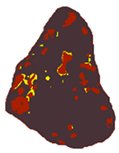	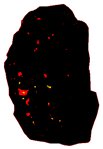	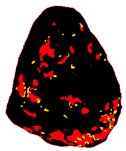
HSVSC VS. Granite	HSVSC VS. Limestone	HSVSC VS. Quartz fine sandstone	HSVSC VS. Basalt

**Table 12:** Two-way ANOVA without replication for the effects of material type on $R_{s}$.

Material Type	SS	DF	MS	F	*p* Value	Partial η^2^
Aggregate	473.698	3	157.89	5.4740	0.01326	0.578
Bitumen	213.714	4	53.42	1.8522	0.18397	0.382

Note: SS = sum of squares; DF = degrees of freedom; MS = mean square; F = F statistic. The analysis was based on 20 combination-level mean stripping ratios, each calculated by averaging the results of six independent specimens. Because one mean value was used for each material combination, the aggregate × bitumen interaction could not be independently separated from the residual variation.

**Table 14:** Three-Way ANOVA for Effect of Image Acquisition Environments on $R_{s}$.

Sequence	Imaging Acquisition Parameters	*p* Value	F Value
1	Light source	0.0004	12.6
2	Background color	<0.0001	57.97
3	Background transparency	<0.0001	99.07
4	Light source & Background color	0.588	0.29
5	Light source & Background transparency	0.913	0.01
6	Background color & Background transparency	<0.0001	82.33
7	Three-way interaction	0.799	0.06

**Table 13:** Comparison of Stripping Performance for Best- and Worst-Case Combinations.

Material Combination	Average $R_{s}$ (%)	Range	Normalized Range	Coefficient of Variation
Quartz fine sandstone + SBS-modified bitumen	1.41	0.26	0.57	0.1844
Granite + emulsified bitumen	30.17	50.97	1.69	0.58

## Data Availability

The original contributions presented in this study are included in the article. Further inquiries can be directed to the corresponding author(s).

## Nomenclature

Abbreviation	Full name
ASTM	American Society for Testing and Materials
FT	Fluorescence tracing
SQ	Stripping quantification
CO	Controlled optical conditions
IQA	Image quality assessment
GT	Ground truth
AA	Adhesion assessment
FTM	Fluorescence tracing mechanism
BAP	Bituminous-coated aggregate particle
UV	Ultraviolet
HSV	Hue-Saturation-Value
RGB	Red-Green-Blue
LAR-IQA	Lightweight no-reference image quality assessment model
MOS	Mean Opinion Score
TTA	Two-mode Threshold Algorithm
HSVSG	Hue-Saturation-Value Segmentation based upon Grayscale
HSVSC	Hue-Saturation-Value Segmentation based upon Color
DC	Dice Coefficient
TP	True Positive
RS	Stripping Ratio
CV	Coefficient of Variation
ANOVA	Analysis of Variance
GRA	Grey Relational Analysis
SBS	Styrene-Butadiene-Styrene
SBR	Styrene-Butadiene Rubber
BBS	Binder Bond Strength
KAN	Kolmogorov–Arnold Network
wt%	Weight percentage
B-U	Black background + UV + opaque background
B-U-T	Black background + UV + transparent background
G-U	Green background + UV + opaque background
G-U-T	Green background + UV + transparent background
B-UN	Black background + UV + Natural light + opaque background
B-UN-T	Black background + UV + Natural light + transparent background
G-UN	Green background + UV + Natural light + opaque background
G-UN-T	Green background + UV + Natural light + transparent background

## Appendix A. Additional Technical Details of Fluorescence Tracer Application, Optical Acquisition, and Image Processing

**Table A1:** Key parameters of the fluorescence tracer, optical acquisition hardware, and image-processing workflow.

Category	Item	Description
Fluorescent tracer	Composition/property	Oil-based fluorescent tracer; previous characterization indicated alkane-like oil as the main component, possibly with a small amount of fluorescent substance
	Solvent/formulation	Oil-based formulation with a tracer-to-diluent ratio of 3:1
	Application method	Uniformly brushed onto the BAP surface using a fine soft brush
	Waiting time before imaging	1 h at room temperature
	Basis for timing	Prior observations showed that fluorescence contrast between bitumen and exposed aggregate became most distinct after approximately 60 min
UV illumination	UV wavelength	365 nm
	UV arrangement	Two UV light sources positioned on both sides of the camera
Imaging hardware	Camera	Hikvision industrial camera (Hangzhou Hikvision Digital Technology Co., Ltd., Hangzhou, China)
	Lens focal length	8 mm
	Exposure time	100,000 ms
	Aperture	f/6
	Working distance	44 cm
	Horizontal UV–camera distance	30 cm
	Illumination level	Approximately 700 lux
HSVSC segmentation	Blue-noise hue range	198–245°
	Purple-noise hue range	245–306°
	Threshold strategy	The blue and purple hue ranges were literature-informed and fixed at 198–245° and 245–306°, respectively. The grayscale threshold of 90 for black-region extraction was empirically determined through preliminary inspection of representative fluorescence images. All thresholds were fixed across the dataset rather than automatically estimated for individual images.

## References

[1] Neupane P., Wu S. (2025). A comprehensive review of moisture damage in asphalt mixtures: Mechanisms, evaluation methods, and mitigation strategies. Constr. Build. Mater..

[2] Xu J.-Y., Ma B., Mao W.-J., Si W., Wang X. (2023). Review of interfacial adhesion between asphalt and aggregate based on molecular dynamics. Constr. Build. Mater..

[3] Tang Y., Fu Z., Raos G., Ma F., Zhao P., Hou Y. (2024). Molecular dynamics simulation of adhesion at the asphalt-aggregate interface: A review. Surf. Interfaces.

[4] Han J., Li B., Ji H., Guo F., Wei D., Cao S., Zhang W., Chen X. (2024). Interfacial adhesion between recycled asphalt binder and aggregates considering aggregate surface anisotropy: A molecular dynamics simulation. Constr. Build. Mater..

[5] Shi B., Liu Q., Gao Y., Wu J., Chen J. (2023). Analysis of the cohesion/adhesion proportion around bitumen-mineral failure interface under tensile loading. Constr. Build. Mater..

[6] Wang S., Du F., Alghamdi S., Feng J., Chen F., Wang Z., Wu C., Xiong H., Liu K., Zheng Y. (2023). Effects of loading rates on interfacial adhesion between aggregates and asphalt binders. Constr. Build. Mater..

[7] Pstrowska K., Gunka V., Sidun I., Demchuk Y., Vytrykush N., Kułażyński M., Bratychak M. (2022). Adhesion in Bitumen/Aggregate System: Adhesion Mechanism and Test Methods. Coatings.

[8] Ignatavicius S., Kavanagh A., Brennan M.J., Colleran D., Sheahan J., Newell S. (2021). Experimental investigation of optimum adhesion properties for anionic emulsions in road maintenance applications. Constr. Build. Mater..

[9] Khasawneh M., Al Oqaily D., Abualia A., Alomari A. (2021). Evaluation of aggregate-binder bond strength using the BBS device for different road materials and conditions. Int. J. Pavement Eng..

[10] Xu F., Nie X., Gan W., E H., Xu P., Cao H., Gong R., Zhang Y. (2023). Moisture Sensitivity Evaluation of the Asphalt Mortar-Aggregate Filler Interface Using Pull-Out Testing and 3-D Structural Imaging. Coatings.

[11] Bionghi R., Shahraki D., Ameri M., Karimi M.M. (2021). Correlation between bond strength and surface free energy parameters of asphalt binder-aggregate system. Constr. Build. Mater..

[12] Zhu S., Peng Y., Kong L., Wang D., Zhu H., Kane M., Ren Z., Li Z. (2025). Multi-scale method for investigating the mechanism of water effect on asphalt/aggregate interface adhesion and the enhancement role of oxides. Energy.

[13] Sun G., Ma T., Jiang Y., Hu M., Lu T., Siwainao D.H.I. (2025). Dual self-repairing enhancement mechanisms of blocked dynamic isocyanate prepolymer on aging degradation and fatigue damages of high-content SBS-modified asphalt. Constr. Build. Mater..

[14] Chomicz-Kowalska A. (2021). A Study of Adhesion in Foamed WMA Binder-Aggregate Systems Using Boiling Water Stripping Tests. Materials.

[15] Xiao R., Polaczyk P., Wang Y., Ma Y., Lu H., Huang B. (2022). Measuring moisture damage of hot-mix asphalt (HMA) by digital imaging-assisted modified boiling test (ASTM D3625): Recent advancements and further investigation. Constr. Build. Mater..

[16] Chen B., Wang X., Li Z., Chen K., Li W., Chen Z., Xiong X. (2025). Spatial distribution characteristics and mechanical properties of artificial coarse aggregates with different morphologies in asphalt mixtures. Case Stud. Constr. Mater..

[17] Esmaeili R., Javadi S., Jamshidi H., Taheri M.E., Sanij H.K. (2022). Stripping propensity detection of HMA Mixes: With focus on image processing method. Constr. Build. Mater..

[18] Tayebali A., Kusam A., Bacchi C. (2018). An Innovative Method for Interpretation of Asphalt Boil Test. J. Test. Eval..

[19] Cui P., Wu S., Xiao Y., Wang F., Wang F. (2019). Quantitative evaluation of active based adhesion in Aggregate-Asphalt by digital image analysis. J. Adhes. Sci. Technol..

[20] Bidgoli M.A., Hajikarimi P., Naderi K., Golroo A., Nejad F.M. (2020). Introducing Adhesion-Cohesion Index to Evaluate Moisture Susceptibility of Asphalt Mixtures Using a Registration Image-Processing Method. J. Mater. Civ. Eng..

[21] Peng Y., Zhao T., Zeng Q., Deng L., Kong L., Ma T., Zhao Y. (2023). Interpretation of stripping at the bitumen–aggregate interface based on fluorescence tracing method. J. Mater. Res. Technol..

[22] Peng Y., Zhao T., Miao J., Kong L., Li Z., Liu M., Jiang X., Zhang Z., Wang W. (2024). Evaluation framework for bitumen-aggregate interfacial adhesion incorporating pull-off test and fluorescence tracing method. Constr. Build. Mater..

[23] Zhao T., Peng Y., Zhang Z., Zhu S., Kong L. (2025). Study on function mechanism of fluorescence tracing method at bitumen-aggregate interface. Constr. Build. Mater..

[24] Zhao T., Zhang Z., Peng Y., Zhu S., Lu C., Shi J., Yang X., Yang M. (2025). Multi-scale investigation of temperature, water intrusion, rubber content, and bitumen aging effects on fluorescence tracing characteristics of bitumen. Constr. Build. Mater..

[25] Zong K., Zhu H., Wu S., Wu D., Pang S., Zhai J., Mao H., Ding Y. (2025). Asphalt and Aggregate Fluorescence Tracing Based on Sensors and Ambient Parameter Optimization. Buildings.

[26] Chen Z., Zhang H., Duan H. (2020). Investigation of ultraviolet radiation aging gradient in asphalt binder. Constr. Build. Mater..

[27] El Hakea A.H., Fakhr M.W. (2023). Recent computer vision applications for pavement distress and condition assessment. Autom. Constr..

[28] Zheng L., Xiao J., Wang Y., Wu W., Chen Z., Yuan D., Jiang W. (2024). Deep learning-based intelligent detection of pavement distress. Autom. Constr..

[29] Shang J., Zhang A.A., Dong Z., Zhang H., He A. (2024). Automated pavement detection and artificial intelligence pavement image data processing technology. Autom. Constr..

[30] Yang X., Zhang J., Liu W., Jing J., Zheng H., Xu W. (2024). Automation in road distress detection, diagnosis and treatment. J. Road Eng..

[31] Jing J., Yang X., Cheng H., Feng X., Zheng H., Brilakis I., Liu J. (2025). A critical review on pavement distress detection using images and point clouds from visual features to geometric modeling. J. Road Eng..

[32] Usamentiaga R., Venegas P., Guerediaga J., Vega L., Molleda J., Bulnes F.G. (2014). Infrared thermography for temperature measurement and non-destructive testing. Sensors.

[33] Vavilov V.P., Burleigh D.D. (2015). Review of pulsed thermal NDT: Physical principles, theory and data processing. NDT E Int..

[34] An Y.-K., Kim J.M., Sohn H. (2014). Laser lock-in thermography for detection of surface-breaking fatigue cracks on uncoated steel structures. NDT E Int..

[35] Koch C., Georgieva K., Kasireddy V., Akinci B., Fieguth P. (2015). A review on computer vision based defect detection and condition assessment of concrete and asphalt civil infrastructure. Adv. Eng. Inform..

[36] Spencer B.F., Hoskere V., Narazaki Y. (2019). Advances in computer vision-based civil infrastructure inspection and monitoring. Engineering.

[37] Ciampa F., Mahmoodi P., Pinto F., Meo M. (2018). Recent advances in active infrared thermography for non-destructive testing of aerospace components. Sensors.

[38] Avanaki N.J., Ghildyal A., Barman N., Zadtootaghaj S., Del Bue A., Canton C., Pont-Tuset J., Tommasi T. (2025). LAR-IQA: A Lightweight, Accurate, and Robust No-Reference Image Quality Assessment Model. Computer Vision-ECCV 2024 Workshops.

[39] Chen B., Zhu L., Kong C., Zhu H., Wang S., Li Z. (2022). No-Reference Image Quality Assessment by Hallucinating Pristine Features. IEEE Trans. Image Process..

[40] Kim S., You J. (2024). Efficient LUT Design Methodologies of Transformation between RGB and HSV for HSV Based Image Enhancements. J. Electr. Eng. Technol..

[41] Ma J., Chen J., Ng M., Huang R., Li Y., Li C., Yang X., Martel A.L. (2021). Loss odyssey in medical image segmentation. Med. Image Anal..

[42] Dice L.R. (1945). Measures of the Amount of Ecologic Association Between Species. Ecology.

[43] Wang T., Liang G., Liu Y. (2025). Analysis of the adhesion failure mechanism of the asphalt-aggregate interface under the action of moisture. Constr. Build. Mater..

[44] Huang Z., Lu R., Fu Z., Li J., Li P., Wang D., Wei B., Zhu W., Wang Z., Wang X. (2023). Investigation of Viscoelastic Properties of Polymer-Modified Asphalt at Low Temperature Based on Gray Relational Analysis. Sustainability.

[45] Külekçi G., Hacıefendioğlu K., Başağa H.B. (2025). Enhancing mineral processing with deep learning: Automated quartz identification using thin section images. Int. J. Miner. Metall. Mater..

